# Coordination complexes constructed from pyrazole–acetamide and pyrazole–quinoxaline: effect of hydrogen bonding on the self-assembly process and antibacterial activity†

**DOI:** 10.1039/d1ra09027e

**Published:** 2022-02-14

**Authors:** Karim Chkirate, Khalid Karrouchi, Hind Chakchak, Joel T. Mague, Smaail Radi, N. N. Adarsh, Weiyang Li, Ahmed Talbaoui, El Mokhtar Essassi, Yann Garcia

**Affiliations:** Laboratory of Heterocyclic Organic Chemistry, Department of Chemistry, Faculty of Sciences, Mohamed V University BP1014 Rabat 10100 Morocco; Laboratory of Analytical Chemistry and Bromatology, Faculty of Medicine and Pharmacy, Mohammed V University in Rabat Morocco khalid.karrouchi@um5s.net.ma; Unités d'Appui Techniques À la Recherche Scientifique (UATRS), Centre National Pour la Recherche Scientifique et Technique (CNRST) Rabat 10000 Morocco; Mohammed First University, Oujda, Faculty of Sciences Oujda, LCAE Oujda Morocco; LCAE, Département de Chimie, Faculté des Sciences, Université Mohamed I BP 524 60 000 Oujda Morocco; School of Chemical Sciences, Mahatma Gandhi University Kottayam 686560 Kerala India; Institute of Condensed Matter and Nanosciences, Molecular Chemistry, Materials and Catalysis (IMCN/MOST), Université catholique de Louvain Place L. Pasteur 1 1348 Louvain-la-Neuve Belgium yann.garcia@uclouvain.be; Laboratoire de Biologie des Pathologies Humaines, Faculté des Sciences, Université Mohammed V de Rabat Morocco

## Abstract

Two mononuclear coordination complexes of *N*-(2-aminophenyl)-2-(5-methyl-1*H*-pyrazol-3-yl)acetamide (L_1_), namely [Cd(L_1_)_2_Cl_2_] (C_1_) and [Cu(L_1_)_2_(C_2_H_5_OH)_2_](NO_3_)_2_ (C_2_) and one mononuclear complex [Fe(L_2_)_2_(H_2_O)_2_](NO_3_)_2_·2H_2_O (C_3_), obtained after *in situ* oxidation of L_1_, have been synthesized and characterized spectroscopically. As revealed by single-crystal X-ray diffraction, each coordination sphere made of two heterocycles is completed either by two chloride anions (in C_1_), two ethanol molecules (in C_2_) or two water molecules (in C_3_). The crystal packing analysis of C_1_, C_2_ and C_3_, revealed 1D and 2D supramolecular architectures, respectively, *via* various hydrogen bonding interactions, which are discussed in detail. Furthermore, evaluation *in vitro* of the ligands and their metal complexes for their antibacterial activity against *Escherichia coli* (ATCC 4157), *Pseudomonas aeruginosa* (ATCC 27853), *Staphylococcus aureus* (ATCC 25923) and *Streptococcus fasciens* (ATCC 29212) strains of bacteria, revealed outstanding results compared to chloramphenicol, a well-known antibiotic, with a normalized minimum inhibitory concentration as low as 5 μg mL^−1^.

## Abstract 2

Two mononuclear coordination complexes of *N*-(2-aminophenyl)-2-(5-methyl-1*H*-pyrazol-3-yl)acetamide (L_1_) and one mononuclear complex, obtained after *in situ* oxidation of L_1_, have been synthesized and characterized spectroscopically.

## Graphical abstract

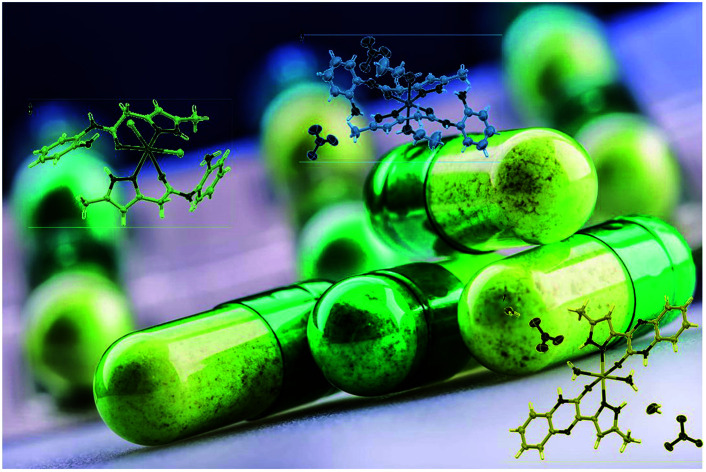

## 1. Introduction

There is growing interest in the development of new active antibacterial compounds as current clinical treatments remain insufficient to meet the challenge of the increasing emergence and spread of antimicrobial resistance.^1^ In Europe, antibiotic resistance is responsible for approximately 33 000 deaths per year.^2^ In the United States, more than 2.8 million people suffer from antibiotic-resistant infections, resulting in more than 35 000 deaths each year.^3^ In 2019, the WHO identified 32 antibiotics in clinical development that address the WHO's list of priority pathogens, of which only six were classified as innovative. Furthermore, a lack of access to quality antimicrobials remains a major issue. Antibiotic shortages are affecting countries of all levels of development and especially in health-care systems.^4^ Therefore, there is an urgent need to develop new antimicrobial agents.

Nitrogen-based ligands are attracting growing attention due to their interesting properties in structural and inorganic chemistry.^5^ Pyrazole derivatives are biologically active heterocyclic compounds.^6–8^ This substance class has been the topic of numerous pharmaceutical studies being used for their medicinal properties such as anti-inflammatory,^9^ antidiabetic,^10,11^ antiviral,^12^ analgesic,^13^ antitumor,^14^ catecholase,^15^ and antimicrobial properties.^16^

On other hand, nitrogen systems have attracted more attention in recent years because of their interesting properties in coordination chemistry.^17–19^ However, many reports on transition metal complexes explain their efficient bioactivity against a range of bacterial and fungal species.^20–22^ In particular, heterocyclic metal complexes dominated medicinal chemistry due to their wide range of properties.^23–25^ Metal complexes tethered with heterocyclic moieties like imidazole, pyrazole, 1,2,4-triazoles and benzimidazole have received remarkable interest as broad spectrum antibacterial, antifungal and antiviral agents.^26–29^ Therefore, the antibacterial activity of many metal complexes has been demonstrated against several bacterial species both *in vitro* and *in vivo*, making it as promising antibacterial agents for use against these bacteria, pending a greater understanding of its safety upon systemic or topical administration in humans.^30–36^ Recently, a study investigated organometallic compounds submitted to the Community for Open Antimicrobial Drug Discovery (CO-ADD) databank, established a classification based on the nature of their metal element, activity, as well as toxicity.^37^ Metal-containing compounds show actually a significantly higher success rate (9.9%) compared to purely organic molecules (0.87%). Out of 906 compounds, 88 show activity against at least one of the tested strains, including fungi, while showing no cytotoxicity against mammalian cell lines or hemolytic properties. Amongst the metal complexes, cadmium, copper and iron, were the most frequent elements found in active ‘non-toxic’ compounds and show the highest overall success rate.^37^

**Fig. 1:**
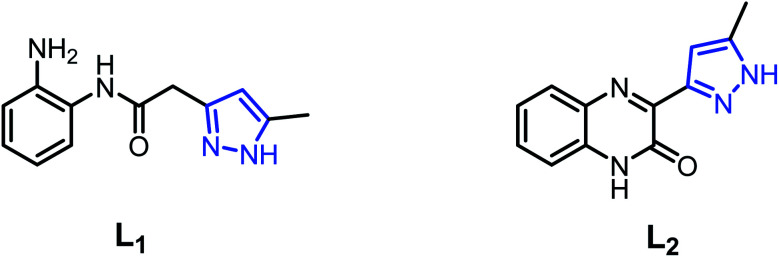
Structures of *N*-(2-aminophenyl)-2-(5-methyl-1*H*-pyrazol-3-yl)acetamide (L_1_) and 3-(5-methyl-1*H*-pyrazol-3-yl)quinoxalin-2(1*H*)-one (L_2_).

In order to search for new ligand candidates for assemblies of metal complexes, we considered the case of pyrazole acetamide ligands with O and N donor atoms.^38–40^ These molecules are particularly interesting as ligands for the construction of polynuclear complexes as models for bioinorganic systems.^41,42^ As a continuation of our research along this line,^43,44^ we have successfully synthesized three new Cd(ii), Cu(ii) and Fe(ii) coordination complexes derived from the ligands, namely *N*-(2-aminophenyl)-2-(5-methyl-1*H*-pyrazol-3-yl)acetamide (L_1_) and 3-(5-methyl-1*H*-pyrazol-3-yl)quinoxalin-2(1*H*)-one (L_2_) (Fig. 1). The molecular structures of the complexes were confirmed by single-crystal X-ray diffraction. All ligands and metal complexes were evaluated *in vitro* for their antibacterial activity against *Escherichia coli* (ATCC 4157), *Pseudomonas aeruginosa* (ATCC 27853), *Staphylococcus aureus* (ATCC 25923) and *Streptococcus fasciens* (ATCC 29212) strains of bacteria.

## 2. Experimental

### 2.1. General methods

Melting points were measured using a Buchi B-545 digital capillary melting point apparatus and used without correction. Reactions were checked with TLC using aluminum sheets with silica gel 60 F254 from Merck. IR spectra were recorded on a PerkinElmer VERTEX 70 FT-IR spectrometer covering field 400–4000 cm^−1^. ^1^H and ^13^C NMR spectra were recorded in DMSO-*d*_6_ on a Bruker spectrometer (300 MHz). Mass spectra were collected using an API 3200 LC/MS/MS system, equipped with an ESI source. Chemical reagents were purchased from Fluka, Sigma and Aldrich chemicals. ^57^Fe Mössbauer spectra were measured in transmission geometry at room temperature with a constant acceleration mode conventional spectrometer equipped with a 50 mCi ^57^Co(Rh) source and a Reuter Stokes proportional counter. A microcrystalline sample of C_3_ was sealed in a Plexiglas sample holder. The spectrum was fitted using Recoil 1.05 Mössbauer Analysis software (Lagarec K., Rancourt D. G.: Recoil, Mössbauer spectral analysis software for Windows 1.0. Department of Physics, University of Ottawa, Canada (1998)). The isomer shift is given with respect to α-Fe at room temperature. X-Ray powder diffraction patterns were recorded on a D8-Advance diffractometer (Bruker, Germany) working with a Cu Kα radiation (*λ* = 1.5148 Å).

### 2.2. Synthesis

#### 2.2.1. Synthesis of (*Z*)-4-(2-oxopropylidene)-4,5-dihydro-1*H*-1,5-benzodiazepin-2(3*H*)-one (3)

A solution of dehydroacetic acid (3.36 g, 0.02 mol) and of *o*-phenylenediamine (4.32 g, 0.04 mol) in xylene (80 mL) was refluxed for 4 h. Next, the precipitated product was filtered under reduced pressure and then recrystallized from ethanol. Yield: 75% (2.52 g); m.p. (°C): 236–238; IR (ATR, *γ* (cm^−1^)): 1671, 1607, 1575; ^1^H NMR (300 MHz, DMSO-*d*_6_, *δ* (ppm)): 2.00 (s, 3H, CH_3_); 3.00 (s, 2H, CH_2_); 5.20 (1H, s); 7.10 (4H, m); ESI-MS: *m*/*z* = 217 [M + H]^+^.

#### 2.2.2. Synthesis of *N*-(2-aminophenyl)-2-(5-methyl-1*H*-pyrazol-3-yl)acetamide (L_1_)

A mixture of (*Z*)-4-(2-oxopropylidene)-4,5-dihydro-1*H*-1,5-benzodiazepin-2(3*H*)-one (2 g, 0.92 mmol) and stoichiometric amount of hydrazine hydrate (0.46 g, 0.92 mmol) in ethanol (40 mL) were refluxed for 2 h. After concentration of the solvent volume to 20 mL, the solution was allowed to stand; the precipitate formed was filtered off and then recrystallized from ethanol. Single crystals were obtained after recrystallization from ethanol. Yield: 80% (1.6 g); m.p. (°C): 170–172; IR (ATR, (cm^−1^)): 3000–3400 (NH, NH_2_), 1737 (C=O), 1655 (C=N); ^1^H NMR (300 MHz, DMSO-*d*_6_, *δ* (ppm)): 2.51 (s, 3H, CH_3_), 2.20 (s, 2H, CH_2_), 4.86 (s, 2H, NH_2_), 5.94 (s, 1H, H_pyrazole_), 6.52–7.16 (m, 5H, H_Ar_), 9.25 (s, 1H, N–H_amide_), 12.24 (s, 1H, NH_pyrazole_); ESI-MS: *m*/*z* = 231 [M + H]^+^.

#### 2.2.3. Synthesis of [Cd(L_1_)_2_Cl_2_] (C_1_)

CdCl_2_·2.5H_2_O (100 mg, 0.44 mmol, 1 eq.) was dissolved in water (10 mL) and added to a solution of L_1_ (200 mg, 0.87 mmol, 2 eq.) in ethanol (15 mL). The resulting light yellow solution was left at room temperature. Colorless column-like single crystals were obtained by slow evaporation of a clear light yellow solution of the reaction mixture after 48 h. Yield = 88% (490 mg); FT-IR (ATR, (cm^−1^)): 3391–3291 (NH, NH_2_), 3117–2866 (CH), 1746 (C=O), 1653 (C=N), 1625, 1604, 1586 (C=C), 608 (Cd–Cl), 541 (Cd–N), 404 (Cd–O); ESI-MS: *m*/*z* = 607.1030 [M − Cl + H]^+^ for C_24_H_28_CdCl_2_N_8_O_2_ in MeOH.

#### 2.2.4. Synthesis of [Cu(L_1_)_2_(C_2_H_5_OH)_2_]·(NO_3_)_2_ (C_2_)

Cu(NO_3_)_2_·3H_2_O (110 mg, 0.46 mmol, 1 eq.) was dissolved in water (10 mL) and added to a solution of L_1_ (200 mg, 0.87 mmol, 2 equiv.) in ethanol (15 mL). The resulting solution was stirred and warmed slightly. The light green precipitate was filtered and then recrystallized from ethanol and left at room temperature. Light green single crystals were obtained by slow evaporation of the reaction mixture after 24 h. Yield: 90% (580 mg); FT-IR (ATR, (cm^−1^)): 3398–3217 (NH, NH_2_), 3121–2905 (CH), 1746 (C=O), 1614 (C=N), 1554, 1499, 1453 (C=C), 427 (Cu–N); ESI-MS: *m*/*z* = 615.2382 [M + H]^+^ for C_28_H_40_CuN_8_O_4_ in MeOH.

#### 2.2.5. Synthesis of [Fe(L_2_)_2_(H_2_O)_2_](NO_3_)_2_·2H_2_O (C_3_)

Fe(NO_3_)_3_·9H_2_O (180 mg, 0.45 mmol, 1 eq.) was dissolved in water (10 mL) and added to a solution of L_1_ (200 mg, 0.87 mmol, 2 eq.) dissolved in ethanol (15 mL). The resulting red solution was stirred and warmed slightly, and left at 10 °C. Red orange single crystals were obtained by slow evaporation of the reaction mixture after 24 h, and filtrated. Yield: 45% (280 mg); FT-IR (ATR, (cm^−1^)): 3198–2860 (CH), 1663 (C=O), 1624 (C=N), 1575, 1537, 1518 (C=C), 505 (Fe–N); ESI-MS: *m*/*z* = 566.1096 [M + Na]^+^ for C_24_H_24_FeN_8_O_4_ in MeOH. When the reaction was carried out at 40 °C, a black powder precipitated which was filtrated and analyzed by X-ray powder diffraction and ^57^Fe Mössbauer spectroscopy. From this black powder, red orange crystals could be isolated, presenting the same structure as C_3_.

### 2.3. X-ray analysis

X-ray single-crystal data were collected on single crystals using Mo Kα (*λ* = 0.7107 A) radiation on a Bruker SMART APEX diffractometer equipped with CCD area detector. Unit cell refinement data reduction (SAINT) and structure solution as well as refinement (SHELXTL)^45^ were carried out using the software package of SMART APEX. The structures of C_1_, C_2_ and C_3_ were solved by direct method and refined in a routine manner. In both structures, non-hydrogen atoms were treated anisotropically. Molecular graphics were generated by using the softwares MERCURY 3.9 (ref. 46) and POV-Ray. The details of the X-ray crystal data and the structure solution as well as the refinement are given in Table 1. CCDC 2095071–2095073 for C_1_, C_2_ and C_3_, respectively contain the supplementary crystallographic data for these compounds.

**Table 1:** Refinement parameters and crystal data for C_1_, C_2_ and C_3_

CCDC number	C_1_	C_2_	C_3_
	2095071	2095072	2095073
**Crystal data**			
Chemical formula	C_24_H_28_CdCl_2_N_8_O_2_	C_28_H_40_CuN_8_O_4_·2(NO_3_)	C_24_H_24_FeN_8_O_4_·2(NO_3_)·2(H_2_O)
*M*_r_	643.84	740.24	704.41
Crystal system, space group	Monoclinic, *Cc*	Triclinic, *P*	Triclinic, *P*
*T* (K)	150	240	150
*a*, *b*, *c* (Å)	13.7075(8), 21.2787(13), 9.1536(6)	9.0258(5), 10.0091(6), 11.2402(6)	8.392(3), 11.420(4), 15.823(6)
*α*, *β*, *γ* (°)	*β*: 95.127(1)	74.769(1), 66.668(1), 80.782(1)	78.679(4), 86.124(4), 80.487(4)
*V* (Å^3^)	2659.2(3)	897.84(9)	1465.5(9)
*Z*	4	1	2
Radiation type	Mo Kα	Mo Kα	Mo Kα
*μ* (mm^−1^)	1.06	0.67	0.60
Crystal size (mm)	0.35 × 0.13 × 0.12	0.27 × 0.12 × 0.11	0.28 × 0.25 × 0.04

**Data collection**			
Diffractometer	Bruker Smart APEX CCD		
Absorption correction	Multi-scan SADABS		
*T*_min_, *T*_max_	0.75, 0.88	0.82, 0.93	0.73, 0.98
No. of measured, independent and observed [*I* > 2*σ*(*I*)] reflections	25873, 7227, 6853	16816, 4438, 3338	12137, 6014, 3364
*R*_int_	0.029	0.031	0.042
(sin *θ*/*λ*)_max_ (Å^−1^)	0.698	0.668	0.629

**Refinement**			
*R* [*F*^2^ > 2*σ*(*F*^2^)], w*R*(*F*^2^), *S*	0.023, 0.053, 1.03	0.045, 0.129, 1.03	0.069, 0.215, 1.00
No. of reflections	7227	4438	6014
No. of parameters	337	241	427
No. of restraints	2	29	
H-atom treatment	H-atom parameters constrained	H-atom parameters constrained	H-atom parameters constrained
Δ*ρ*_max_, Δ*ρ*_min_ (e Å^−3^)	1.09, −0.24	0.58, −0.22	1.35, −0.45
Absolute structure	Refined as an inversion twin		
Absolute structure parameter	0.267(16)		

### 2.4. Antibacterial activity

The antibacterial activity of the synthesized compounds was determined according to the method described in our previous work.^43^

## 3. Result and discussion

### 3.1. Synthesis of pyrazole–acetamide ligands L_1_

**Scheme 1:**
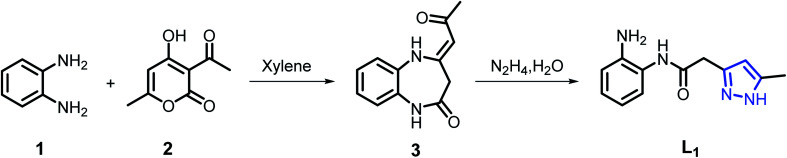
Synthetic route for preparation of L_1_.

Our strategy was to develop a simple, high-yield, synthetic procedure in a few steps to prepare the desired acetamide derivative. The development of the synthesis of L_1_ is given in Scheme 1. The major product 3^47^ was produced in good yield by condensation of *o*-phenylenediamine with dehydroacetic acid (DHA) in refluxing xylene for 4 h. The second step consists in the condensation of a stoichiometric amount of hydrazine monohydrate with the benzodiazepine compound 3 in refluxing ethanol for 2 h to give the ligand pyrazole–acetamide L_1_ in good yield^48^ (Scheme 1).

### 3.2. Synthesis of coordination complexes C_1_, C_2_ and C_3_

The three coordination complexes [Cd(L_1_)_2_Cl_2_] (C_1_), [Cu(L_1_)_2_(C_2_H_5_OH)_2_](NO_3_)_2_ (C_2_) and [Fe(L_2_)_2_(H_2_O)_2_](NO_3_)_2_·2H_2_O (C_3_) were obtained as single crystals after recrystallization from ethanol during the reaction carried out in an aqueous ethanolic solution involving pyrazole acetamide L_1_ and Cd(ii), Cu(ii) and Fe(iii) (metal/ligand ratio 1 : 2), respectively (Table 2).

**Table 2:** Hydrogen-bond geometry (Å, °) of C_1_, C_2_ and C_3_^a^

D–H⋯A	D–H	H⋯A	D⋯A	D–H⋯A	Symmetry codes
C_1_					
N1–H1A⋯Cl2	0.91	2.57	3.469(3)	169	*x* − 1/2, −*y* + 3/2, *z* − 1/2
N1–H1B⋯Cl1	0.91	2.52	3.259(3)	139	*x*, *y*, *z*
N2–H2A⋯Cl2	0.91	2.61	3.280(3)	131	*x*, *y*, *z* − 1
N4–H4A⋯N5	0.91	2.06	2.954(4)	168	*x*, *y*, *z*
N5–H5A⋯Cl2	0.91	2.59	3.318(3)	137	*x*, *y*, *z*
N5–H5B⋯Cl1	0.91	2.59	3.473(3)	165	*x* + 1/2, −*y* + 3/2, *z* + 1/2
N6–H6A⋯O2	0.91	2.29	3.186(4)	170	*x*, −*y* + 1, *z* + 1/2
N8–H8A⋯N1	0.91	2.04	2.937(4)	171	*x*, *y*, *z*
C10–H10⋯Cl1	0.95	2.74	3.617(3)	154	*x* + 1/2, −*y* + 3/2, *z* − 1/2
C4–H4⋯Cg2	0.95	2.89	3.587(4)	131	*x*, *y*, *z* − 1
C22–H22⋯Cg5	0.95	2.82	3.6939(3)	144	*x*, −*y* + 1, *z* + 1/2

C_2_					
N1–H1A⋯O2	0.91	2.17	3.062(3)	166	−*x* + 1, −*y* + 2, −*z* + 1
N1–H1B⋯O3	0.91	2.12	3.028(6)	173	*x* − 1, *y*, *z*
N2–H2A⋯O2	0.91	1.96	2.857(2)	167	*x*, *y*, *z*
N4–H4A⋯N1	0.91	2.05	2.958(2)	173	−*x* + 1, −*y* + 1, −*z* + 1
C5–H5⋯O4	0.94	2.54	3.431(8)	158	−*x* + 1, −*y* + 2, −*z* + 2
C10–H10⋯O4	0.94	2.39	3.329(4)	173	−*x* + 2, −*y* + 2, −*z* + 1
C12–H12B⋯O3	0.97	2.46	3.388(6)	161	−*x* + 2, −*y* + 1, −*z* + 1
O5–H5A⋯O2	0.87	2.17	2.898(2)	141	*x*, *y* − 1, *z*
O5–H5A⋯O4	0.87	2.45	3.262(5)	156	*x*, *y* − 1, *z*

C_3_					
O3–H3A⋯N1	0.87	2.00	2.855(4)	168	−*x* + 1, −*y* + 1, −*z* + 1
O3–H3B⋯O10	0.87	1.89	2.708(11)	157	*x*, *y*, *z*
O3–H3B⋯N10	0.87	2.52	3.381(17)	173	*x*, *y*, *z*
O4–H4A⋯O12	0.87	1.80	2.638(5)	162	*x*, *y*, *z*
O4–H4B⋯N5	0.87	1.99	2.856(4)	174	−*x* + 1, −*y* + 1, −*z*
N2–H2A⋯O7	0.91	1.91	2.805(4)	166	*x*, *y* + 1, *z*
N3–H3C⋯O11	0.91	2.02	2.917(4)	169	*x*, *y* − 1, *z*
N6–H6A⋯O11	0.91	1.94	2.845(4)	173	*x*, *y* − 1, *z*
N7–H7C⋯O7	0.91	1.98	2.885(4)	176	*x*, *y* + 1, *z*
C14–H14⋯O5	0.95	2.54	3.400(5)	175	*x* + 1, *y*, *z*
C24–H24A⋯O9	0.98	2.49	3.47(3)	173	−*x* + 1, −*y* + 2, −*z*
O11–H11A⋯O6	0.87	2.05	2.853(4)	153	*x* + 1, *y* + 1, *z*
O11–H11B⋯O9	0.87	1.76	2.61(2)	167	*x*, *y*, *z*
O12–H12D⋯O5	0.87	1.96	2.814(6)	168	*x*, *y*, *z*
O12–H12D⋯N9	0.87	2.57	3.359(6)	151	*x*, *y*, *z*
O12–H12E⋯O9	0.87	2.01	2.87(14)	170	*x*, *y* − 1, *z*

a C1: Cg2 and Cg5 are, respectively, the centroids of the N7/N8/C32/C22/C21 and C1⋯C6 rings.

**Scheme 2:**
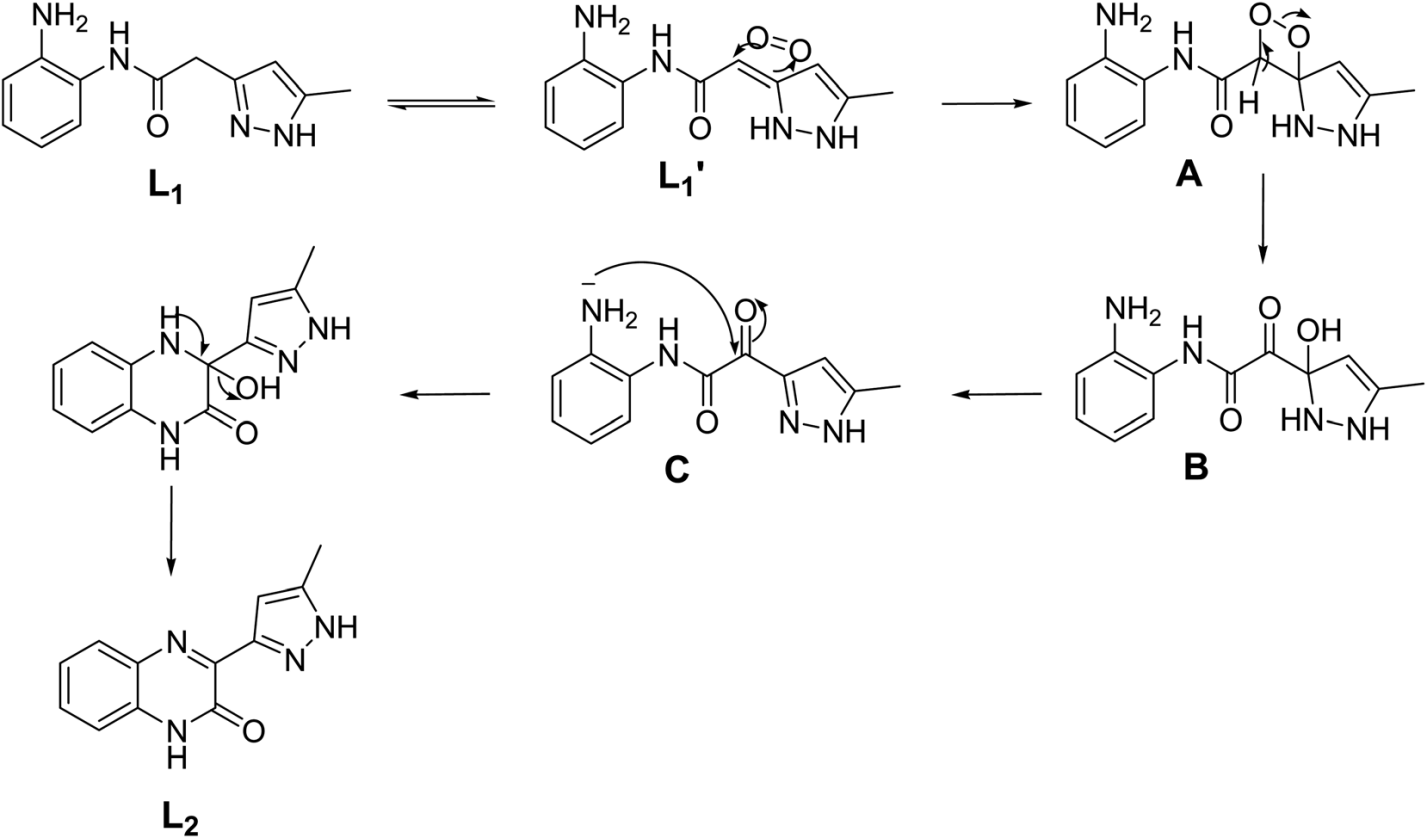
Plausible reaction mechanism for the formation of L_2_.

While classic coordination occurred for L_1_ with cadmium and copper, an unexpected oxidation reaction followed by an intramolecular cyclization of the formed intermediate was presumably observed when iron nitrate was used as chelating agent. Thus, the iron(ii) complex C_3_ was synthesized in low yield by the reaction of Fe(NO_3_)_3_·9H_2_O with L_1_ in a 1 : 2 molar ratio using ethanol as a solvent. The low yield of the compound is attributed to the involvement of the L_1_ ligand in the redox reaction with Fe(iii) ion, where the Fe(iii) ion was reduced to Fe(ii) ion. The Fe(ii) ion produced was complexed with the ligand L_2_ formed by the oxidation of L_1_ (Scheme 2). The first Fe(iii) promoted *in situ* oxidation of a thiazoline-2-thione to the corresponding hetero-disulphide with concomitant coordination to Fe(ii) was reported by Raper *et al.*,^49^ Several studies have been conducted mainly to characterize the products of reactions between nitrogen-containing ligands and Fe(iii) ions under aerobic conditions.^50–53^ The presence of the electron-withdrawing groups increases the reduction potential of the Fe^3+^ + e^−^ ↔ Fe^2+^ redox couple, making the reduction product thermodynamically more stable.^54^

A plausible mechanism (Scheme 2) is proposed to explain the original transformation of pyrazole acetamide L_1_ into the new ligand L_2_. Thus, the participation of the ligand L_1_ in redox reaction with Fe(iii) ion, where Fe(iii) ion was reduced to Fe(ii) ion and L_1_ was oxided to L_1_′, is proposed. Afterwards, air oxygen reacted with the carbon–carbon double bond of the tautomeric form L_1_′ affording spiro oxetane pyrazole A which undergoes a ring opening of the oxetane moiety under the effect of a base to lead to the hydroxy pyrazoline B. The latter compound aromatizes by the loss of a water molecule to give the ketonic amide C which undergoes an intramolecular cyclization to afford after a loss of a water molecule pyrazolyl quinoxaline acting as coordination compound towards Fe(ii) ion produced by reduction of Fe(iii). It should be noted that a similar oxidation reaction has already been observed in our previous work on 1,2,4-triazolo pyrimidines.^43^

### 3.3. Description of the crystal structures of the complexes [Cd(L_1_)_2_Cl_2_] (C_1_), [Cu(L_1_)_2_ (C_2_H_5_OH)_2_](NO_3_)_2_ (C_2_) and [Fe(L_2_)_2_(H_2_O)_2_](NO_3_)_2_·2H_2_O (C_3_)

**Fig. 2:**
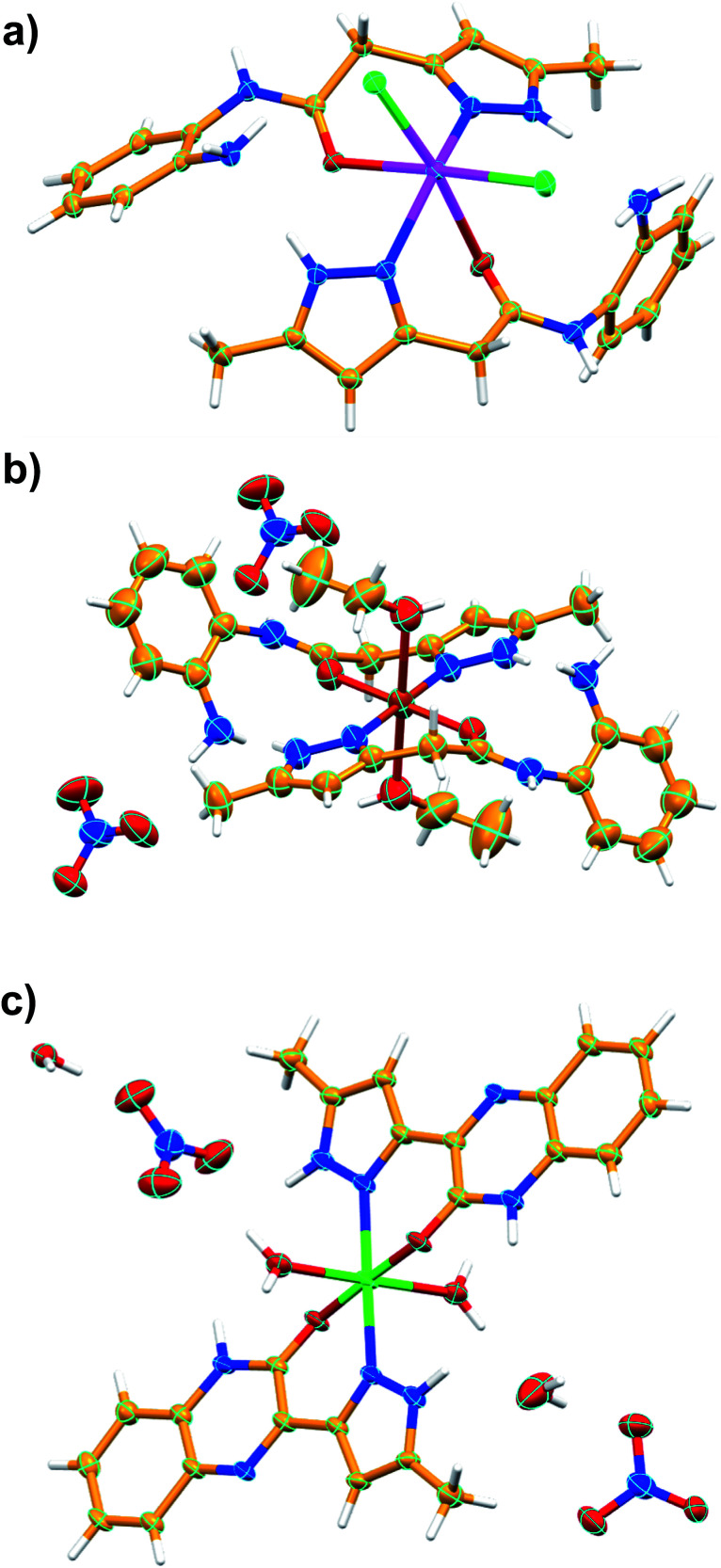
Asymmetric unit of C_1_ (a), C_2_ (b) and C_3_ (c). Color codes: C – orange, N – blue, H – white, Cd – Magenta, Cu – dark orange, Fe – green, O – red, Cl – green.

The crystal structures of C_1_, C_2_ and C_3_ are shown in Fig. 2. Crystallization of all the three coordination compounds was obtained by reaction of the ligand L_1_ (for C_1_ and C_2_) or L_2_ (for C_3_) and metal salts in aqueous ethanolic solution (metal : ligand = 1 : 2) by slow evaporation.

#### 3.3.1. [Cd(L_1_)_2_Cl_2_] (C_1_)

**Fig. 3:**
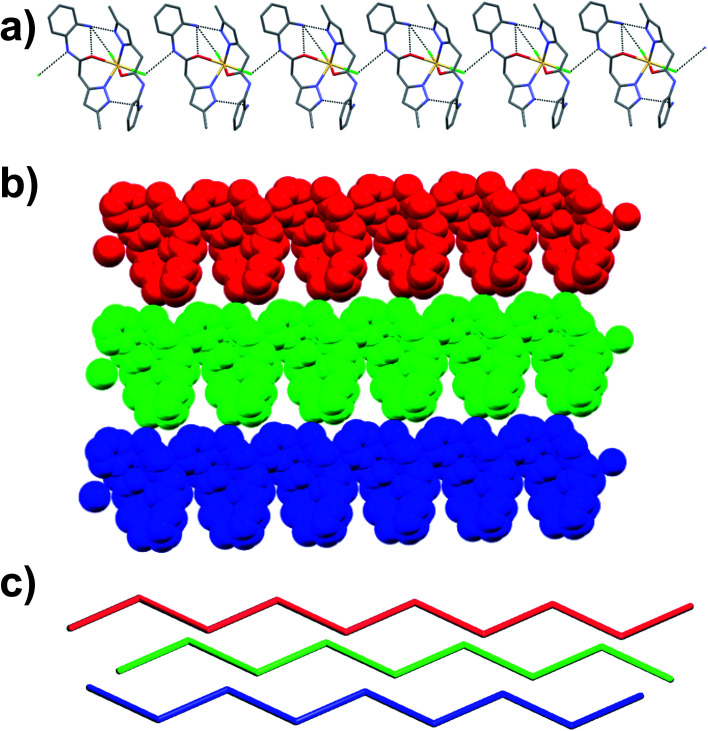
Crystal structure illustration of C_1_, (a) 1D hydrogen bonded chains (black dotted lines represent the N–H⋯Cl interactions), (b) parallel packing of 1D chains (view along crystallographic axis ‘*b*’), (c) TOPOS view of the parallel packing of 1D chains.

Colorless column-like single crystals of C_1_ crystalized in the monoclinic space group *Cc* (Table 1). The asymmetric unit contains one crystallographically unique Cd(ii) ion, two molecules of ligand L_1_ and two chloride anions. The Cd(ii) metal center showed distorted octahedral geometry [∠N–Cd–Cl = 100.78(7)–106.73(7)°; ∠N–Cd–O = 77.13(8)–78.18(8)°; ∠Cl–Co–Cl = 100.22(3)°; ∠O–Cd–O = 86.72(10)°; ∠O–Cd–Cl = 83.69(6)°] in which the coordination sites are occupied by two chloride anions, and N atoms of pyrazole and O atoms of amide of the ligand L_1_. The equatorial coordination sites of Cd(ii) are occupied with the two N atoms and one O atom of the pyrazole and amide functionalities of the ligand L_1_, respectively, and one Cl^−^ anion, and the axial position are coordinated to one Cl^−^ anion and one O atom of amide moiety of L_1_. Basically, the non-planar ligand L_1_ [torsion angle between the planes of the aromatic rings = 47.84(2) and 60.33(8)° for crystallographically independent ligands] coordinates to Cd(ii) in an N, O–chelating mode, generating the six-membered chelate ring as expected. Such chelation was strongly supported by strong intramolecular hydrogen bonding involving amino group of L_1_ with the metal bound Cl^−^ anion [N–H⋯Cl = 3.259(3) Å, ∠N–H–Cl = 139°], O atom of amide functionality of L_1_ [N–H⋯O = 2.934(3) Å, ∠N–H–O = 109°] and N atom of pyrazole [N–H⋯N = 2.937(4) Å, ∠N–H–N = 171°] resulted in a four-membered, six-membered and five-membered rings, respectively. Moreover, supramolecular assembly in the crystal structure of C_1_ is mainly driven by N–H⋯Cl hydrogen-bonding interactions comprising N–H of the amide moiety of L_1_ and metal bound Cl^−^ anion [N–H⋯Cl = 3.280(3) Å, ∠N–H–Cl = 131°], resulting in a one-dimensional (1D) hydrogen bonded chain structure. Such chains are further assembled in parallel fashion with the support of various van der Waals interactions (Fig. 3).

#### 3.3.2. [Cu(L_1_)_2_(C_2_H_5_OH)_2_](NO_3_)_2_ (C_2_)

**Fig. 4:**
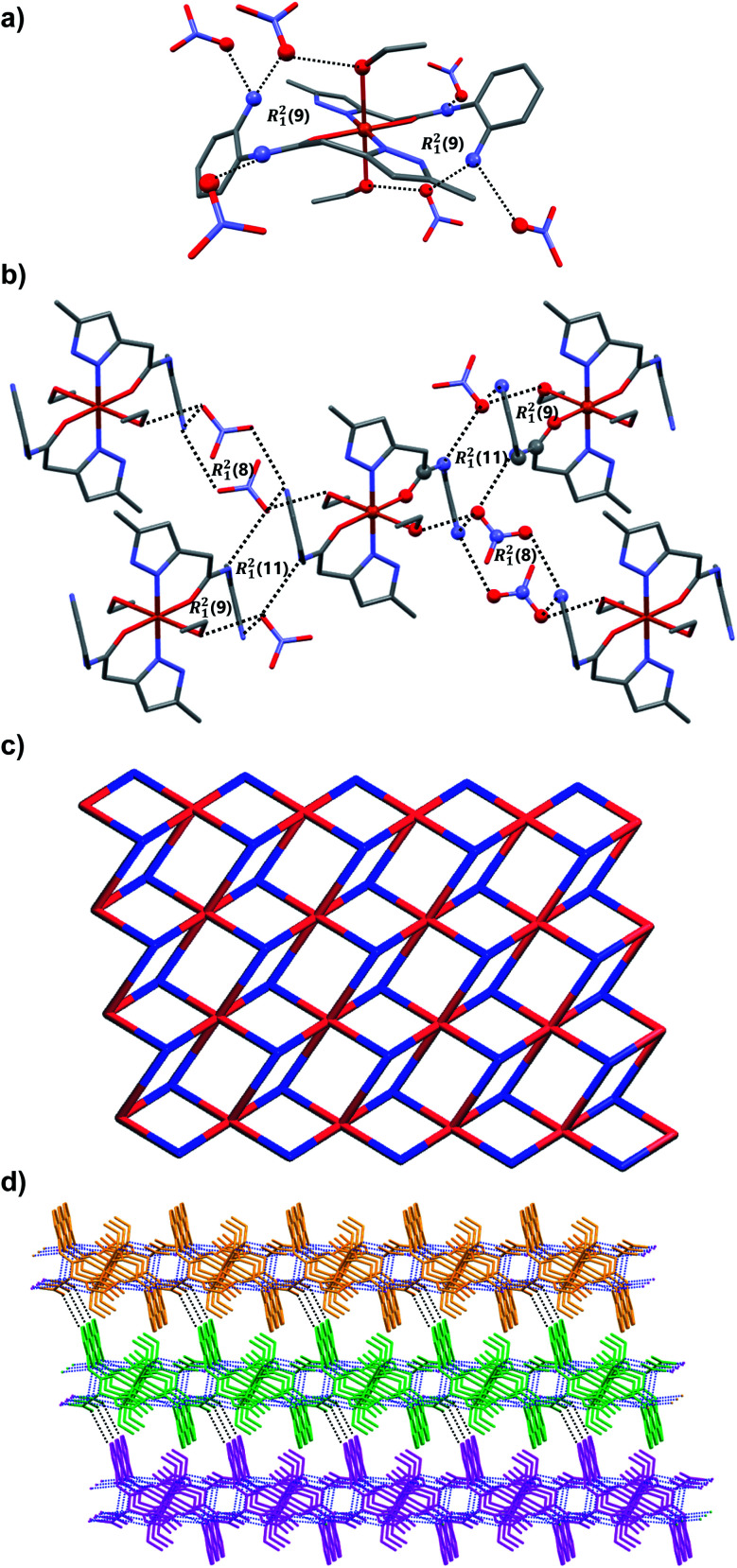
Crystal structure illustration of C_2_, (a) the N–H⋯O and O–H⋯O hydrogen bonding in C2 displaying the *R*^2^_1_(9) ring, (b) various hydrogen bonding and the view of *R*^2^_1_(11), *R*^2^_1_(9) and *R*^2^_1_(8) rings, (c) TOPOS view of 2D hydrogen bonded sheet, (d) the stacking of 2D layers along crystallographic axis ‘*a*’ (adjacent 2D layers are shown in orange, green and magenta).

Light green colored single crystals of C_2_ belong to triclinic centrosymmetric space group *P*1̄. The asymmetric unit contains one-half of Cu(ii) ion, one full molecule of ligand (coordinated to the metal center Cu(ii) through N atom of pyrazole and O atom of amide functionality of L_1_ in a chelate fashion), one molecule of ethanol (also coordinated to Cu^2+^) and one nitrate counter anion. Thus the entire octahedral coordination complex is generated by the inversion center of symmetry located on the metal ion Cu^2+^. The Cu(ii) metal center showed slightly distorted octahedral geometry [∠N–Cu–O = 89.48(7)–90.52(7)°; ∠O–Cu–O = 89.40(6)–90.60(6)°] in which the equatorial coordination positions of Cu(ii) are occupied by the O and N atoms of L_1_ through its amide and pyrazole moieties resulted in N,O-chelated six membered ring, and axial positions of the metal center are coordinated to the O atoms of ethanol (EtOH). As expected the ligand showed nonplanar geometry, revealed from the torsion angle [68.62(6)°] between the planes of the aromatic rings. The amine and amide N–H of the coordination complex form hydrogen bonding with O atoms of the nitrate anion [N–H⋯O = 3.028(7) Å, ∠N–H–O = 173°]; the nitrate anion also involved in bifurcated hydrogen bonding with N–H of amine [N–H⋯O = 2.857(2) Å, ∠N–H–O = 167°] and O–H of metal bound ethanol molecule [O–H⋯O = 2.898(2) Å, ∠O–H–O = 141°] resulted in *R*^2^_1_(9) ring (Fig. 4a). Supramolecular assembly in the crystal structure of C_2_ is mainly directed by intermolecular N–H⋯O and O–H⋯O hydrogen-bonding interactions involving the nitrate anion with amide, metal bound ethanol and amine resulted in three synthons having graph-sets of *R*^2^_1_(11), *R*^2^_1_(9) and *R*^2^_1_(8) (Fig. 4b). Self-assembly through these three synthons lead to the formation of a two-dimensional (2D) hydrogen bonded sheet structure. If the discrete [Cu(L_1_)_2_(EtOH)_2_] complex, and nitrate anions are taken as nodes, 2D hydrogen bonded network in this coordination compound can be simplified to a 2D (3,6)-connected kgd net with a point (Schläfli) symbol of {4^3^}_2_{4^6^·6^6^·8^3^} (Fig. 4c). Such 2D layers are stack up each other along crystallographic axis ‘*a*’ with the support of C–H⋯O hydrogen bonding involving aromatic C–H and O atom of nitrate anion [C–H⋯O = 3.431(8) Å, ∠C–H–O = 158°] (Fig. 4d).

#### 3.3.3. [Fe(L_2_)_2_(H_2_O)_2_](NO_3_)_2_·2H_2_O (C_3_)

**Fig. 5:**
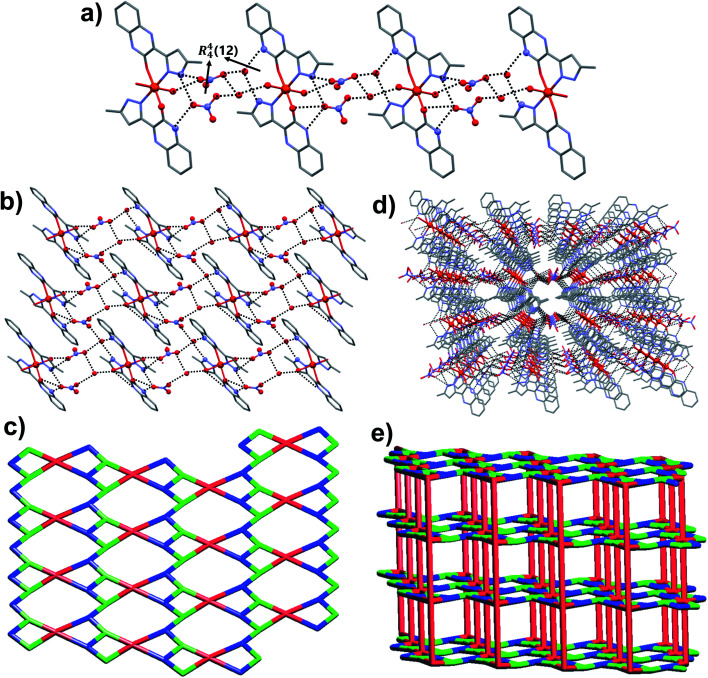
Crystal structure illustration of C_3_, (a) 1D chain self-assembly of C_3_ through *R*^4^_4_(12) rings *via* various hydrogen bonding, (b) 2D hydrogen bonded assembly, (c) TOPOS view of 2D hydrogen bonded network, (d) 3D hydrogen bonded network, (e) TOPOS view of 3D [3^2^·6-c]-connected net.

Orange colored single crystals of C_3_ got crystalized in the centrosymmetric triclinic space group *P*1̄ (Table 1). The asymmetric unit comprises of one Fe(ii) ion, two molecules of ligand L_2_, two water molecules (both water and L_2_ are coordinated to Fe(ii)), two nitrate counter anions and two solvated water molecules. The Fe(ii) metal center showed distorted octahedral geometry [∠O–Fe–O = 88.91(11)–91.62(11)°; ∠O–Fe–N = 84.56(11)–97.20(11)°] in which the equatorial and axial coordination sites are occupied by ligand L_2_ and water molecules, respectively. In the crystal structure, the ligand L_2_ showed slightly non-planar structure in which the torsion angle between the plane of quinoxalinone and pyrazole is 11.43–13.11°. The O atom of quinoxalinone and N atom of pyrazole moieties of L_2_ are coordinated to the Fe(ii) resulted in a N,O-chelated six membered ring. The hydrogen bonding interactions of nitrate anions with solvated water molecule [N–H⋯O = 2.885(5) Å, ∠N–H–O = 177°; O–H⋯O = 2.813(5)–2.87(3) Å, ∠O–H–O = 168–170°] and nitrate⋯π interaction [3.454(6) Å] of nitrate with pyrazole ring of metal coordinated L_2_ resulted in an twelve membered ring which contains the four donor and acceptor atoms with a graph-set symbol *R*^4^_4_(12) (Fig. 5a). This synthon is connected to the discrete [Fe(L_2_)_2_(H_2_O)_2_] complex unit node through nitrate anions, metal bound water molecule and quinoxalinone moiety *via* N–H⋯O and O–H⋯O interactions [N–H⋯O = 2.805(4) Å, ∠N–H–O = 166°; O–H⋯O = 2.708(12) Å, ∠O–H–O = 157°]. The same synthon is further assembled with the neighboring [Fe(L_2_)_2_(H_2_O)_2_] complex unit node through another twelve membered ring having a graph-set of *R*^4^_4_(12) N–H⋯O [N–H⋯O = 2.845(4) Å, ∠N–H–O = 173°] and O–H⋯O [O–H⋯O = 2.61(3)–2.87(3) Å, ∠O–H–O = 162–170°] hydrogen bonding involving nitrate anions, solvated water molecules, quinoxalinone N–H and Fe(ii) coordinated water molecule (Fig. 5a). The hydrogen bonding interactions through these two synthons resulted in the self-assembly of C2 along crystallographic axis “*b*” lead to the formation of one-dimensional hydrogen bonded chain (Fig. 5a). The self-assembly further follow through these two twelve membered ring synthons along crystallographic axis “*a*” resulted in a 2D hydrogen bonded sheet network structure (Fig. 5b and c). Interestingly, such 2D network structure further self-assembled through O–H–N hydrogen bonding [O–H⋯N = 2.856(4) Å, ∠O–H–N = 174°] involving metal bound water and N atom of quinoxalinone (along crystallographic axis “*c*”) lead to the formation of a 3D hydrogen bonded network (Fig. 5d). If the discrete [Fe(L_2_)_2_(H_2_O)_2_] complex, and twelve membered ring synthons are taken as nodes, the 3D hydrogen bonded network can be simplified to a 3D [3^2^·6-c]-connected net having point (Schläfli) symbol of {4·8^2^}_2_{4^2^·6}_2_{4^2^·8^10^ × 10^3^} (Fig. 5e).

### 3.4. Hirshfeld surface analysis

**Fig. 6:**
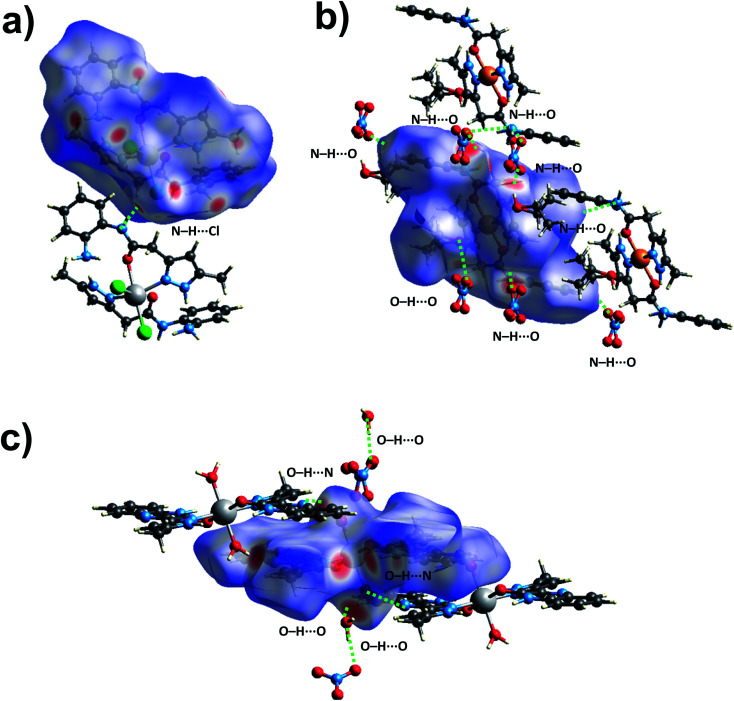
The *d*_norm_ Hirshfeld surfaces of C_1_ (a), C_2_ (b), and C_3_ (c) displaying hydrogen bonding interactions.

To further explore the supramolecular interactions in the crystal structure of the coordination compounds, we have constructed their Hirshfeld surface and 2D-fingerprint plots by using Crystal Explorer program.^55^ The surface where the electron density *ρ*_int_(*r*) of the molecules is larger than the electron density *ρ*_ext_(*r*) of the adjacent molecules is called the Hirshfeld surface.^56^ Hirshfeld surfaces (HS) of the coordination compounds C_1_, C_2_ and C_3_ are shown in Fig. 6, displaying the surface map over the normalized contact distance (*d*_norm_) in which the red and white colors indicating strong proximity and intermediate closeness of atoms to the HS from outside, respectively. We have used the following equation to calculate *d*_norm_ from the values of *d*_e_ (distance between the Hirshfeld surface and external molecule), *d*_i_ (distance between the Hirshfeld surface and inside molecule) and van der Waals radii of the atoms (*r*^vdw^_i_ or *r*^vdw^_e_). From the value of *d*_norm_, we can easily determine the regions participating in the intermolecular interactions in the complexes.
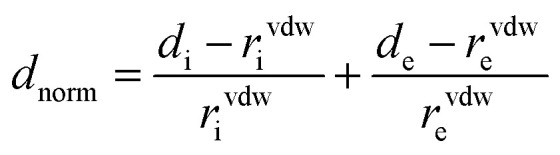


In other words, these two colors indicating strong and intermediate hydrogen bonding interactions present between HS and neighboring atoms outside, respectively. The blue color regions in the HS meaning the longer distances than the van der Waals radii. The HS of C1 was generated by using a standard (high) surface resolution with 3D *d*_norm_ surfaces mapped to a range −0.3180 to 1.5585 a.u. From the *d*_norm_ mapping, it is revealed that strong N–H⋯Cl hydrogen bonding interaction (between amide moiety of L_1_ and chloride anion) was present in the crystal structure of C_1_, as observed from the bright red spots on the HS. The 3D *d*_norm_ surfaced mapping of C_2_ was done within the range of −0.5718 to 1.7268 a.u which showed bright red spots at amine and amide N–H (due to strong N–H⋯O hydrogen bonding with nitrate counter anion), and metal bound water molecule (O–H⋯O hydrogen bonding with nitrate). In the case of complex C_3_, the 3D *d*_norm_ surfaced mapping (range of −0.7131 to 1.2768 a.u.) showed red spots near to metal bound water molecule and quinoxalinone moiety (due to strong O–H⋯O and O–H⋯N hydrogen bonding). We have also plotted the shape index and curvedness of the coordination complexes by using Crystal Explorer program; the red concave surface surrounded by the receptors and blue convex surface surrounding receptors on the HS in the shape index of the coordination complexes further confirm the presence of such hydrogen bonding (Fig. S13 and S14,† ESI).

**Fig. 7:**
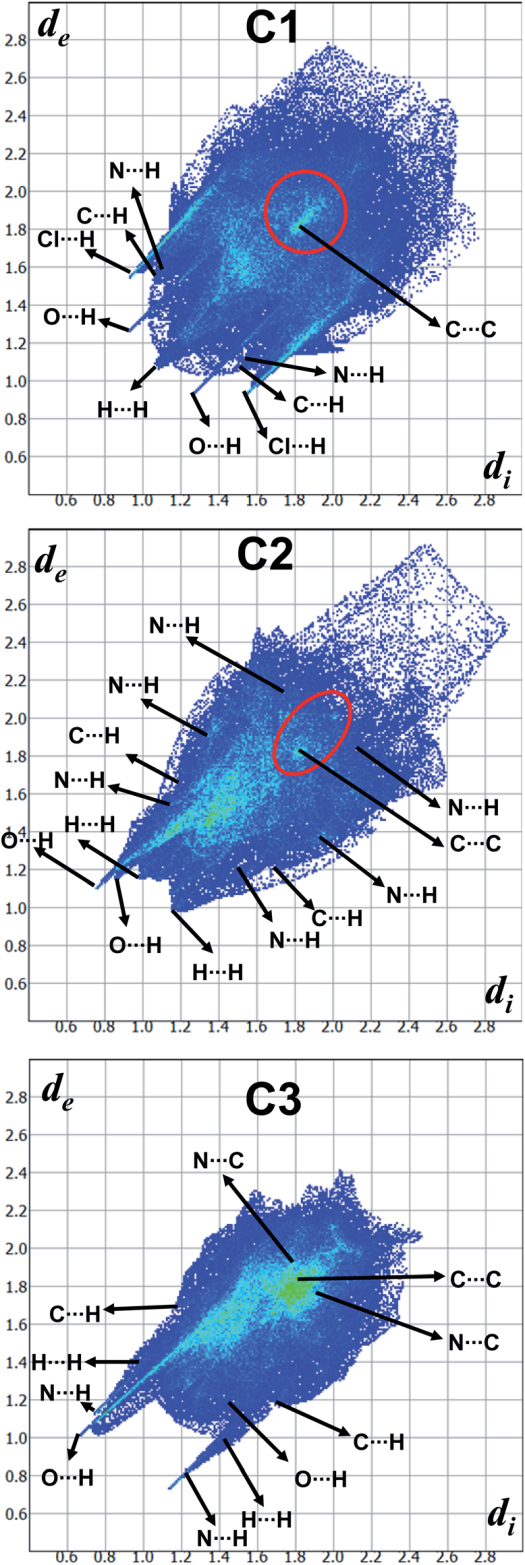
2D Fingerprint plots derived from the Hirshfeld surfaces displaying various intermolecular interactions.

In order to quantify the contribution of various supramolecular interactions in the coordination complexes C_1_, C_2_ and C_3_, we have plotted their 2D fingerprints by using Crystal Explorer program (Fig. 7 and S15–S17,† ESI). The internal *d*_i_ and external *d*_e_ distances between the HS and atom contacts are given in Å. We found that two strong hydrogen bonding N–H⋯O and N–H⋯Cl are presented in the crystal structure of C_1_ which corroborated well with their 2D fingerprints; meaning the contributions of the interatomic contacts to the HF such as Cl⋯H/H⋯Cl (16.1%) and O⋯H/H⋯O (7.3%). Moreover, other weak interatomic contacts to the HF of C1 such as N⋯H/H⋯N (4.5%), C⋯H/H⋯C (17.6%), H⋯H/H⋯H (49.6%) and C⋯C/C⋯C (2.9%) are also present in the 2D fingerprint. Similarly, the main contributions of the compounds C_2_ and C_3_ to the HS are assigned to the close contacts of O⋯H/H⋯O (25% for C_2_ and 25.5% for C_3_), N⋯H/H⋯N (2.3% for C_2_ and 6.7% for C_3_), C⋯H/H⋯C (13.9% for C_2_ and 10% for C_3_), H⋯H/H⋯H (57.5% for C_2_ and 40.5% for C_3_), C⋯C/C⋯C (1.3% for C_2_ and 8.8% for C_3_) and N⋯C/N⋯C (6.7% for C_2_). The interatomic contacts such as O⋯H/H⋯O, N⋯H/H⋯N, C⋯H/H⋯C, H⋯H/H⋯H, C⋯C/C⋯C present in the 2D fingerprints of C_2_ and C_3_ corroborate well with the supramolecular interactions such as N–H⋯O, C–H⋯N, C–H⋯π, other van der Waals interactions and C–H⋯π/π⋯π stacking, respectively present in their crystal structures (Fig. 7).

### 3.5. Mössbauer spectroscopy

**Fig. 8:**
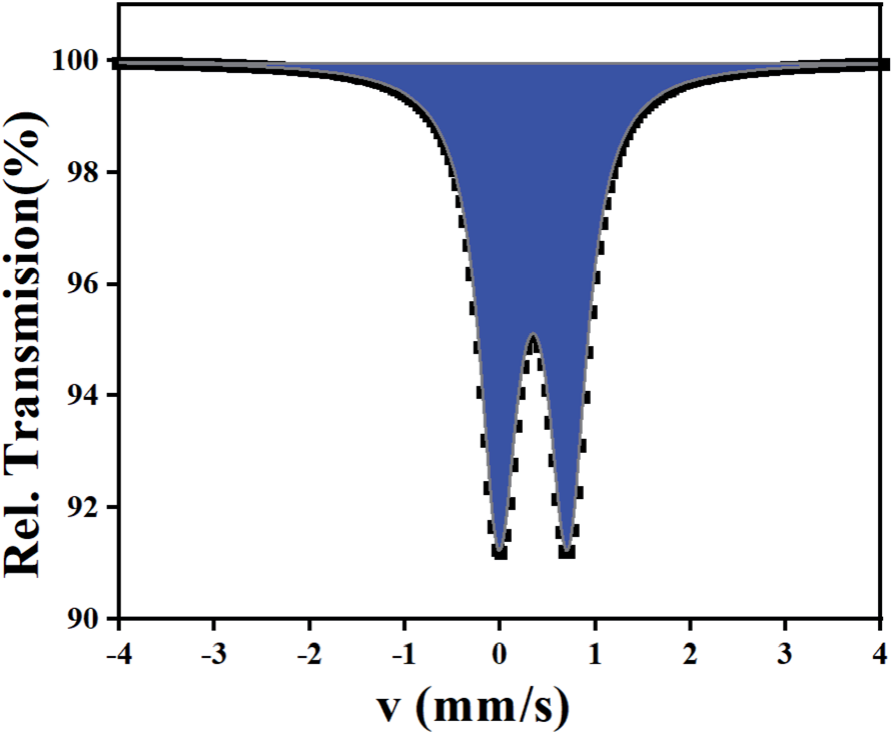
^57^Fe Mössbauer spectrum of C_3_ recorded at 298 K. The half width of the lines *Γ*/2 = 0.23(1) mm s^−1^.

**Fig. 9:**
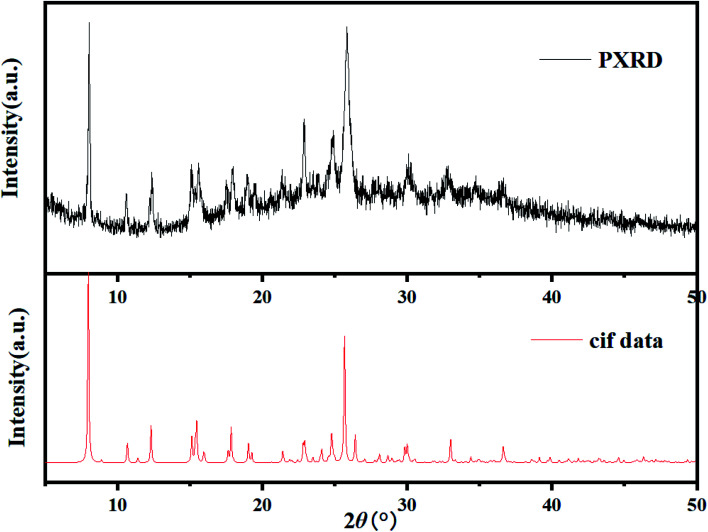
XRPD pattern of the black powder issued from the synthesis of C_3_ compared to the computed XPRD pattern obtained from the cif file of C_3_.

A powdered sample of C_3_ was recorded at 298 K. The spectrum shows a quadrupole doublet with isomer shift *δ* = 0.346(3) mm s^−1^ and quadrupole splitting Δ*E*_Q_ = 0.72(1) mm s^−1^ (Fig. 8). Such a doublet is characteristic of high-spin Fe(iii) species. Measurements were also recorded at high velocity up to *v*_max_ = 10 mm s^−1^ but no oxides were detected. This result contrasts with the one offered by single crystal X-ray diffraction which revealed Fe(ii) species only. A microscope analysis shows that the powder contains few orange single crystals, whereas the majority of the powder is black. A crystal cell parameters analysis of orange crystals revealed similar parameters as those given in Table 1. This result is confirmed by powder X-ray diffraction of the powdered sample which shows an amorphous pattern plus diffraction peaks. These diffraction peaks correspond to the simulated ones from the cif file of C_3_ (Fig. 9). Worth to note that the amount of C_3_ detected in the powder was evaluated below the detection limit of Mössbauer spectroscopy, *i.e. ca.* 2%.

### 3.6. Antibacterial activity

The antibacterial activities of the synthesized molecules (3 and L) and metal complexes (C_1_, C_2_ and C_3_) were tested against *E. coli* and *P. aeruginosa* as Gram-negative and *S. aureus* and *S. fasciens* as Gram-positive microorganisms by the diffusion method disk. Table 3 reports the minimum inhibitory concentration (MIC) which is the lowest concentration for which no growth is detected for 24 h at 37 °C. The results were compared with a standard, chloramphenicol, an antibiotic, *e.g.* used for the treatment of eyelid infection,^57^ at various concentrations.

**Table 3:** Antibacterial activity of ligand and complexes (MIC, μg mL^−1^)

Compound	MIC (μg mL^−1^)			
	*E. coli*	*P. aeruginosa*	*S. aureus*	*S. fasciens*
3	10	10	20	5
L_1_	20	20	20	20
C_1_	5	5	5	10
C_2_	5	10	5	20
C_3_	20	5	10	20
Chloramphenicol	6.25	6.25	12.5	6.25

Overall the three complexes showed higher antibacterial activities against the four strains tested, compared to 3 and L_1_ ligand, except in the case of *S. fasciens* with a remarkable MIC = 5 μg mL^−1^ for 3 (Table 3). Such antibacterial activity of the C_1_–C_3_ complexes compared to the ligand, could be due to the coordination of cadmium, copper and iron metal ions to the condensed ring system (as shown by single crystal X-ray diffraction), thus increasing the delocalization of π electrons throughout the chelated ring and improving the lipophilicity of the complexes and thus the penetration of the complexes into the lipid membrane and further limiting the multiplicity of microorganisms, following Overtone's concept on cell permeability,^58^ and Tweedy's chelation theory.^59^

Remarkably, C_1_ reveals an outstanding activity against both Gram-negative (*E. coli* and *P. aeruginosa*) and Gram-positive (*S. aureus*) bacteria, compared to C_2_, C_3_ complexes, with a MIC value of 5 μg mL^−1^. This value is even better compared to chloramphenicol, a well-known antibiotic. Similarly, C_2_ reveals an identical MIC for *E. coli* and *S. aureus*, whereas C_3_ show excellent activity against *P. aeruginosa* (MIC = 5 μg mL^−1^) and very good activity towards *S. aureus.*

Most excitingly, superior values are found compared to earlier literature reports on Cd(ii), Cu(ii) and Fe(ii) complexes (Table 4).

**Table 4:** Antibacterial activity of C_1_, C_2_, C_3_ and other model complexes

Metal complexes	Antibacterial activity (MIC in μg mL^−1^)			
	*E. coli*	*P. aeruginosa*	*S. aureus*	Ref.
C_1_	5	5	5	This work
C_2_	5	10	5	This work
C_3_	20	5	10	This work
[CuCl_2_(Fpy)_2_]^a^	310	—	150	60
Cu(2,5-xil)_2_^b^	—	31.2	31.2	61
Cu(3,5-xil)_2_^b^	—	31.2	<15.6	62
[Cu(Ligand)_1_(bipy)]ClO_4_·H_2_O^c^	—	—	125	62
[Cu(Ligand)_2_(bipy)]ClO_4_·bipy^c^	—	—	62	62
[Cu(Ligand)_3_(bipy)]ClO_4_·3H_2_O^c^	—	—	62	62
[Cu(tL)_2_](BF_4_)^d^	256	256	—	63
Cu(bcmpo)^e^	64	64	64	64
Cu(bcmp)^e^	64	64	64	64
[Fe(C_18_H_14_O_2_N_10_)Cl]Cl_2_	64	64	32	65
[Fe(C_18_H_14_O_2_N_10_)(NO_3_)](NO_3_)_2_	128	>128	64	65
Cu(naph1pp)_2_^f^	256	512	64	66
Cu(naph2pp)_2_^f^	256	256	256	66
Cu(dansyl)_2_^f^	>512	512	512	66
[Cu(HL_1_)(CH_3_CH_2_OH)](CH_3_COO)^g^	128	128	32	67
[{CdCl(HATtsc)}2(*m*-Cl)_2_]·2H_2_O^h^	25	>100	25	68
[{Cd(NO_3_)(HATtsc)}2(*m*-NO_3_)_2_]^h^	25	>100	25	68
[Cd_3_(bmesal)_2_(Cl)_2_]^i^	140	—	—	69
[Cd(dbnu)]^j^	125	—	125	70
[Cu(dbnu)]^j^	250	—	250	70
[Cu(pdbpo)(NO_3_)(H_2_O)_2_]^k^	256	256	128	71
[Cu(pdbpo)(SCN)(H_2_O)_2_]^k^	512	256	64	71
[Cu(pdbpo)(ClO_4_)(H_2_O)_2_]^k^	512	256	128	71
Cu(DL_1_)^l^	>32	—	>32	72
Fe(L_G_)^m^	>512	>512	512	73
Fe(L_p_)^n^	>512	>512	256	73

a Fpy = 2-fluoropyridine.

b 2,5-xil = 2,5-dimethylaniline, 3,5-xil = 3,5-dimethylaniline.

c bipy = 2,2′-bipyridine; ligand = reaction between meso-1,2-diphenyl-1,2-ethylenediamine with salicylaldehyde (1), 5-bromosalicylaldehyde (2) or 3-methoxysalicylaldehyde (3).

d tL = reaction of 2-aminofluorene with 2-pyridinecarboxaldehyde.

e bcmpo = 3-bis(3′-carboxyl-5′-methyl-1′-pyrazolyl)propan-2-ol; bcmp = 1,3-bis(3′-carboxyl-5′-methyl-1′-pyrazolyl), 2-methyl propane.

f naph1pp = 3-hydroxy-2-methyl-1-naphthyl-4-pyridinonate; naph2pp = 1-(*N*-naphthylcarbamoylpropyl)-3-hydroxy-2-methyl-4-pyridinonate; dansylpp = 2-(*N*-dansylaminomethyl)-3-hydroxy-1,6-dimethyl-4-pyridinonate.

g HL_1_ = *N*,*N*′-bis(salicylidene)diethylenetriamine.

h HATtsc = 2-acetyl-2-thiazoline thiosemicarbazone.

i bmesal = *N*,*N*′-bis(3-methoxysalicylidenimino)-1,3-diaminopropane.

j dbnu = 1,3-diethyl-1,3-bis(4-nitrophenyl)urea.

k pdbpo = 1-phenyl-2,3-dimethyl-4-(*N*-2-hydroxy-4-methoxy-benzaldehyde)-3-pyrazolin-5-one.

l DL_1_ = *N*,*N*′-bis(1-(4-hydroxy-6-methyl-2-oxo-2*H*-pyran-3-yl)ethylidene)malonohydrazide.

m L_G_ = 2-(((2-hydroxynaphthalen-1-yl)methylene)amino)e andthane-1,1-diol.

n L_p_ = 2-(((2-hydroxynaphthalen-1-yl)methylene)amino)-2-phenylethane-1,1-diol.

## 4. Conclusions

In conclusion, three new Cd(ii), Cu(ii) and Fe(ii) complexes formulated as [Cd(L_1_)_2_Cl_2_] (C_1_), [Cu(L_1_)_2_(C_2_H_5_OH)_2_](NO_3_)_2_ (C_2_) and [Fe(L_2_)_2_(H_2_O)_2_](NO_3_)_2_·2H_2_O (C_3_) have been synthesized and their crystal structures have been studied. Crystal structure and Hirshfeld Surface analysis have shown that the crystal lattices of all the complexes are influenced for the presence of several intermolecular interactions, including hydrogen bonds. The N–H⋯O and N–H⋯Cl hydrogen bonding interactions benefits to C_1_, which is the first coordination complex with L_1_, to assemble to form a 1D chain as a primary supramolecular architecture. On the other hand, in complex C_2_, N–H⋯O and O–H⋯O hydrogen bonding play a role to self-assemble the crystallographically independent molecules of complexes and ethanol molecules to form a 2D corrugated hydrogen bonded sheet. The layers are joined by inversion-related C–H⋯O hydrogen bonds. On another hand in complex C_3_, the iron ion is coordinated by two chelating organic ligands and two water molecules with a slightly distorted octahedral geometry. O–H⋯O, N–H⋯O and O–H⋯N hydrogen bonds and π-stacking interactions form layers of cations, anions and solvent water molecules. These are further linked into the full three-dimensional structure by additional hydrogen bonds. Furthermore, the results of antibacterial activity testing reveal that C_1_, C_2_ and C_3_ complexes showed notable activity against all four strains of bacteria studied. Thus, the best result was shown by C_1_, against *E. coli*, *P. aeruginosa* and *S. aureus* bacterial strains, with a remarkable MIC of 5 μg mL^−1^.

## Conflicts of interest

The authors declare no competing financial interest.

## Supplementary Material

RA-012-D1RA09027E-s001

RA-012-D1RA09027E-s002
