# Supplementary material for: Coordination complexes constructed from pyrazole–acetamide and pyrazole–quinoxaline: effect of hydrogen bonding on the self-assembly process and antibacterial activity
Source: RSC Adv. 2022 Feb 14;12(9):5324–39. doi: 10.1039/d1ra09027e (PMC8981392; doi:10.1039/d1ra09027e)
Supplement: RA-012-D1RA09027E-s001 [file RA-012-D1RA09027E-s001.pdf]

Electronic Supplementary Material (ESI) for RSC Advances.
This journal is © The Royal Society of Chemistry 2022

SUPPORTING INFORMATION

Coordination complexes constructed from pyrazole-acetamide and pyrazole-quinoxaline: Effect of hydrogen bonding on the self-assembly process and antibacterial activity

Karim Chkirate,[a] Khalid Karrouchi,[b,*] Hind Chakchak,[c] Joel T. Mague,[d] Smaail Radi,[e] N. N. Adarsh,[f] Weiyang Li,[g] Ahmed Talbaoui,[h] El Mokhtar Essassi,[a] and Yann Garcia[g,*]

[a] Laboratory of Heterocyclic Organic Chemistry, Department of Chemistry, Faculty of Sciences, Mohamed V University, BP1014, Rabat, 10100, Morocco.

[b] Laboratory of Analytical Chemistry and Bromatology, Faculty of Medicine and Pharmacy, Mohammed V University in Rabat, Morocco.

[c] Unités d'Appui Techniques À la Recherche Scientifique (UATRS), Centre National Pour la Recherche Scientifique et Technique (CNRST), Rabat, 10000, Morocco.

[d] Department of Chemistry, Tulane University, New Orleans, LA 70118, USA.

[e] LCAE, Département de Chimie, Faculté des Sciences, Université Mohamed I, BP 524, 60 000 Oujda, Morocco.

[f] School of Chemical Sciences, Mahatma Gandhi University, Kottayam, 686560, Kerala, India.

[g] Institute of Condensed Matter and Nanosciences, Molecular Chemistry, Materials and Catalysis (IMCN/MOST), Université catholique de Louvain, Place L. Pasteur 1, 1348 Louvain-la-Neuve, Belgium.

[h] Laboratoire de Biologie des Pathologies Humaines, Faculté des Sciences, Université Mohammed V de Rabat, Morocco.

*Corresponding authors:
yann.garcia@uclouvain.be (Y. Garcia)
khalid.karrouchi@um5s.net.ma (K. Karrouchi)

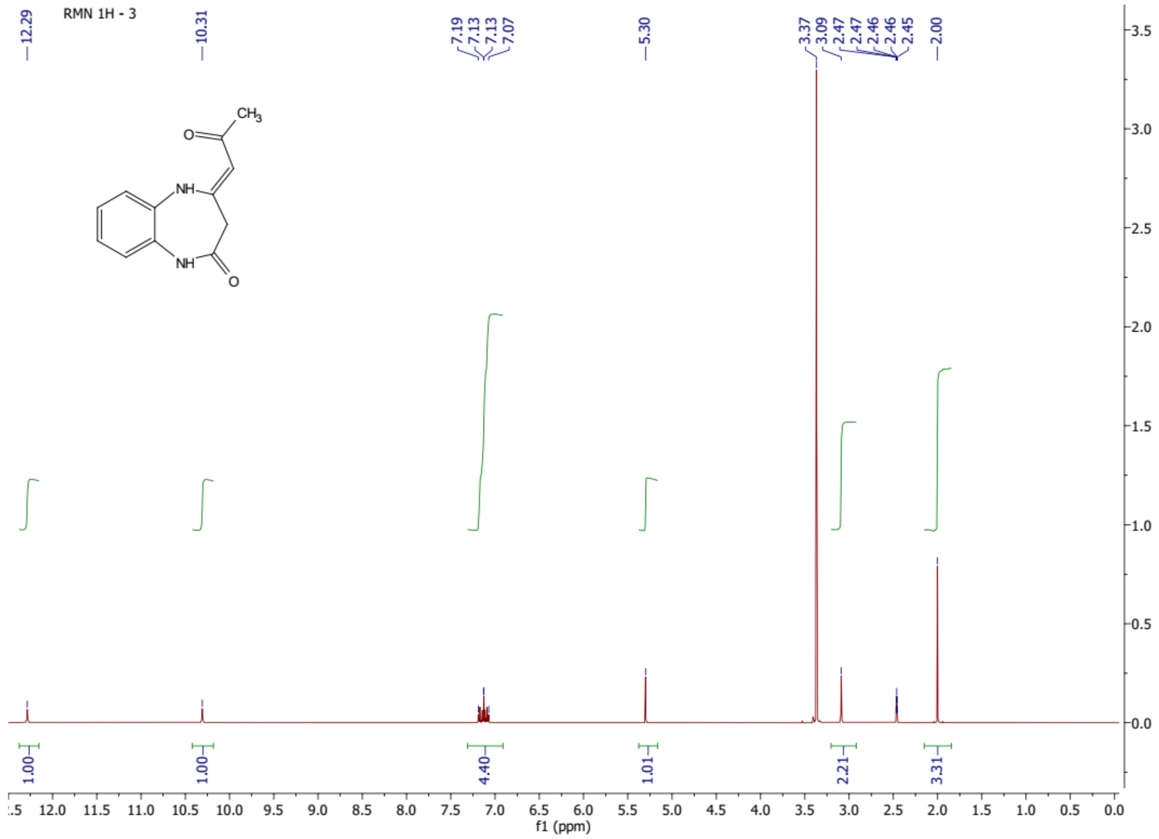

Figure S1. ^{1}H NMR spectrum of ***3***.

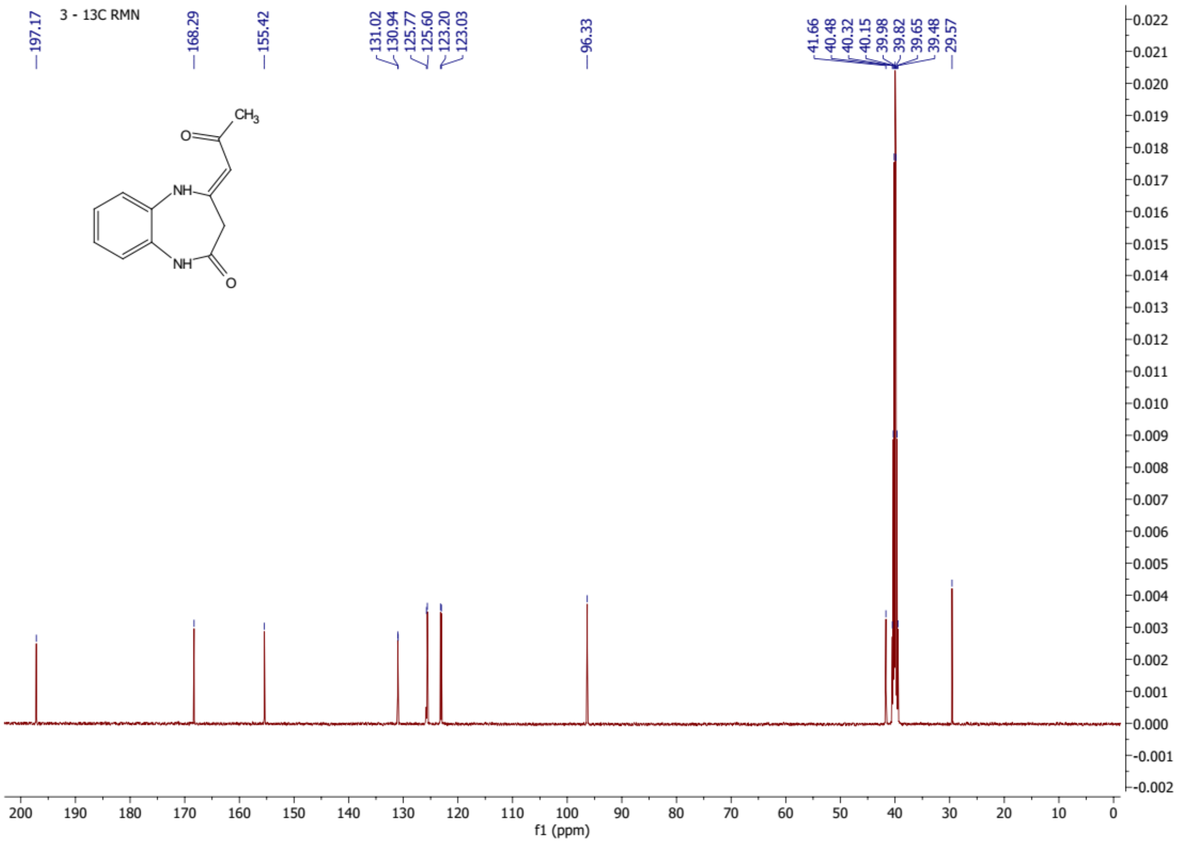

Figure S2. ^{13}C NMR spectrum of ***3***.

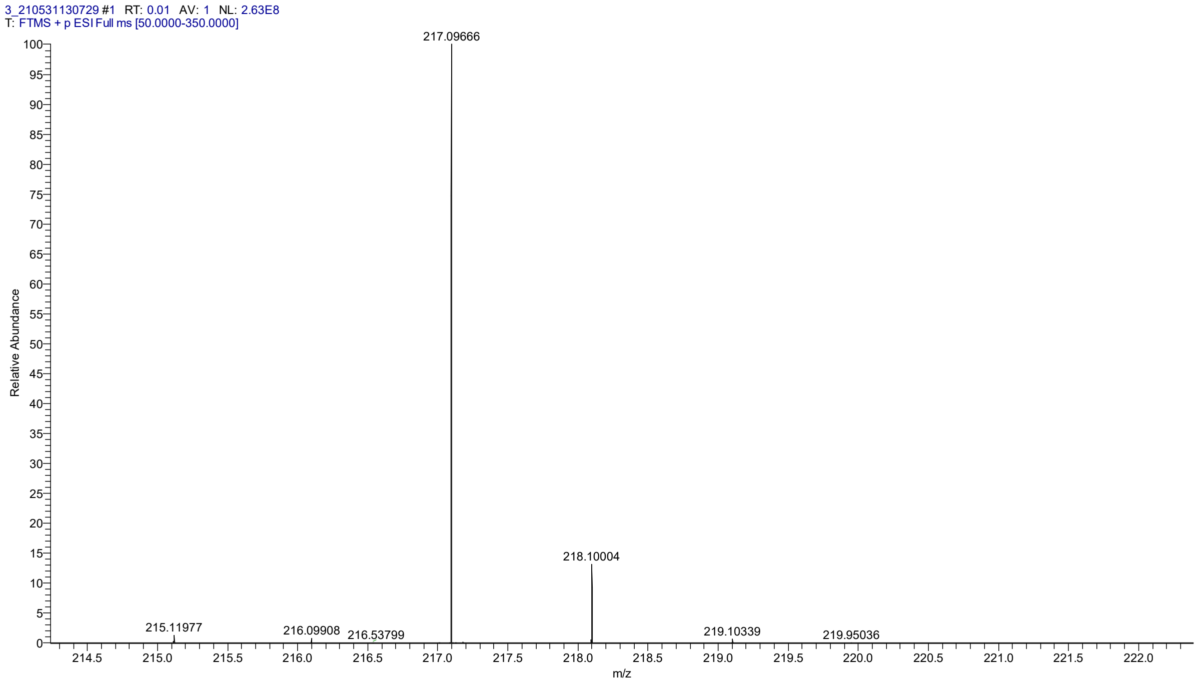

Figure S3. ESI-HRMS spectrum of ***3***.

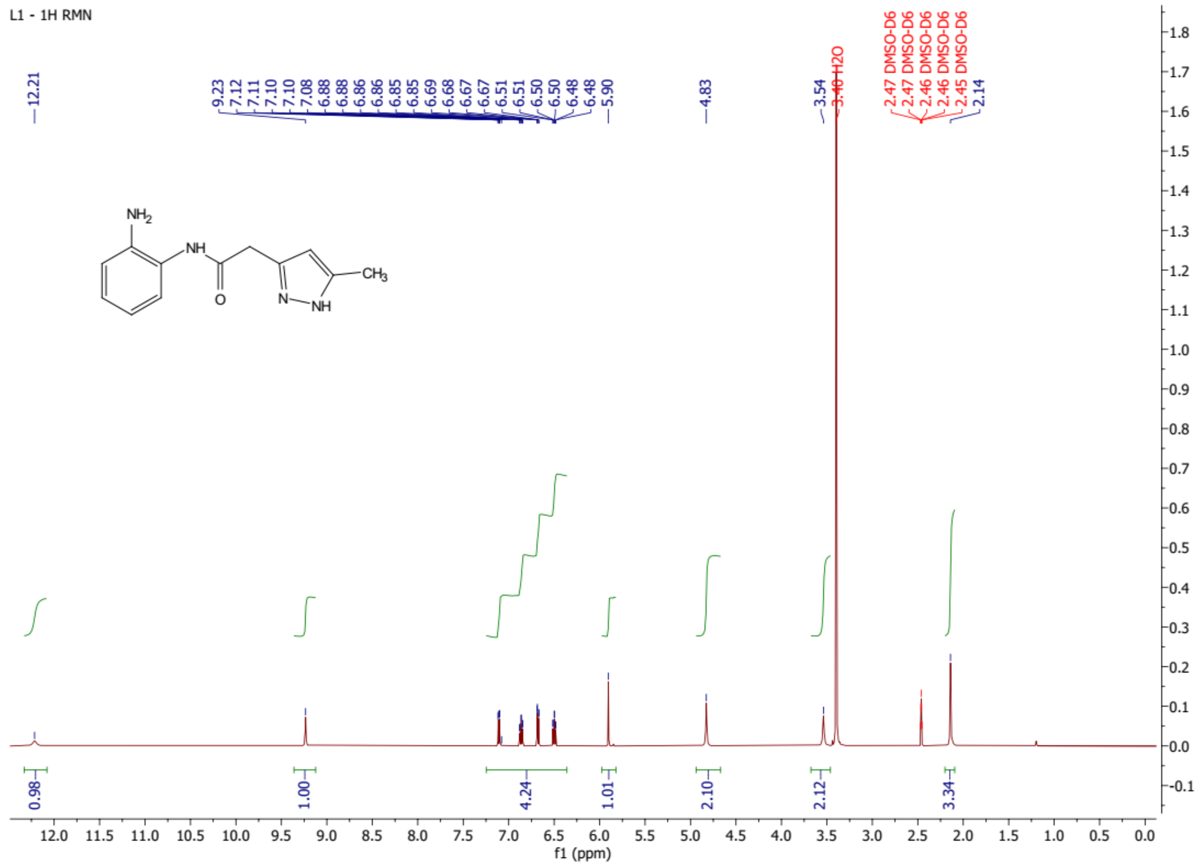

Figure S4. ^{1}H NMR spectrum of ***L1***.

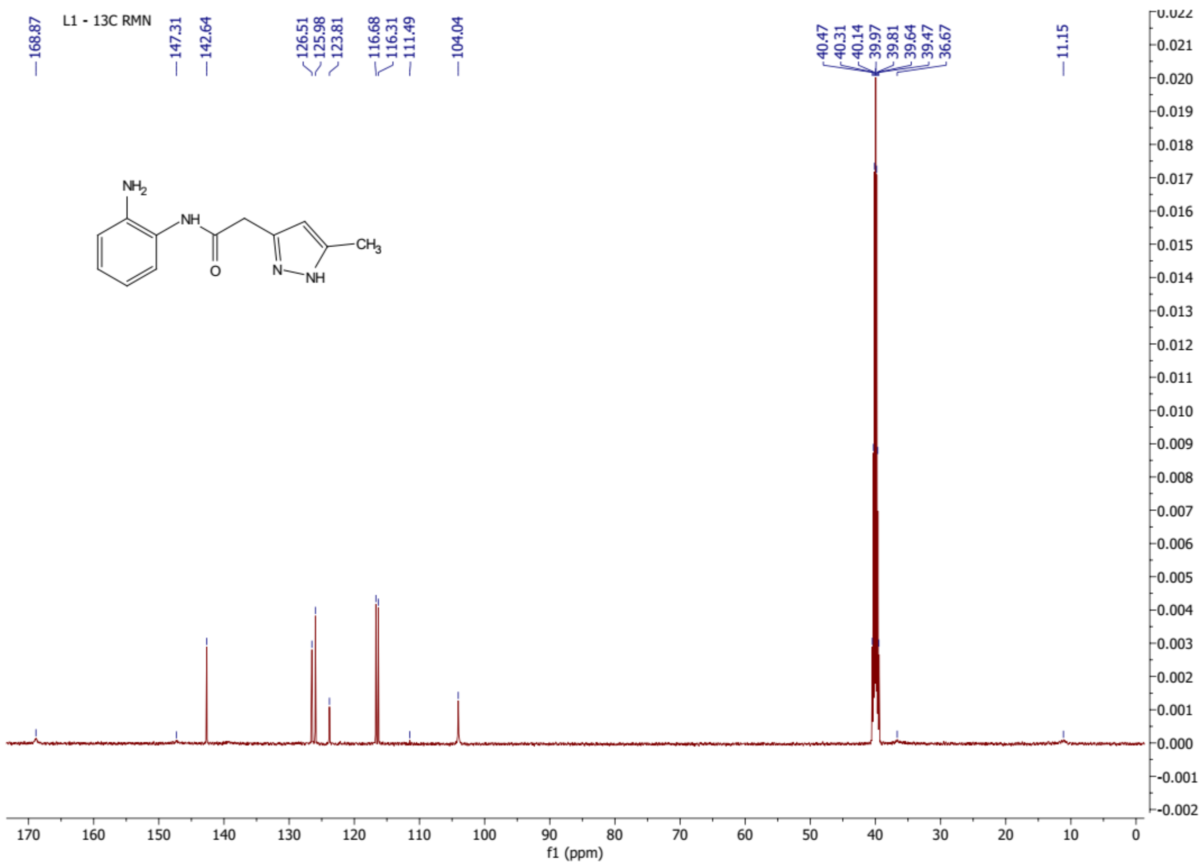

Figure S5. ^{13}C NMR spectrum of ***L1***.

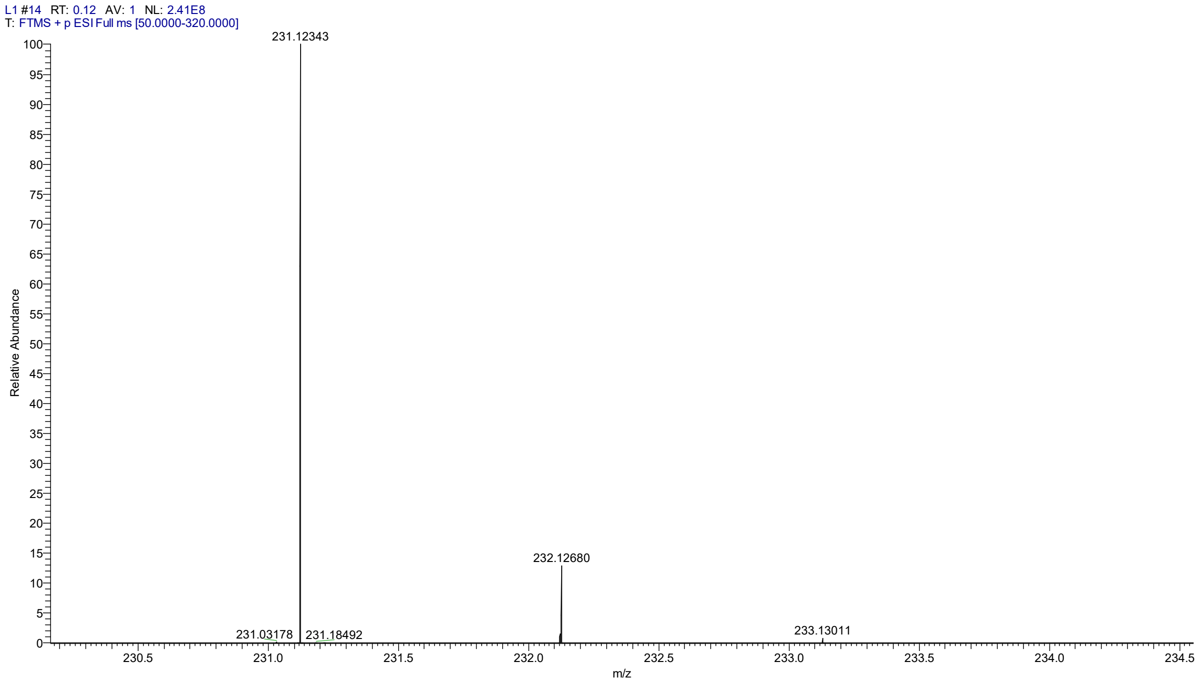

Figure S6. ESI-HRMS spectrum of ***L1***.

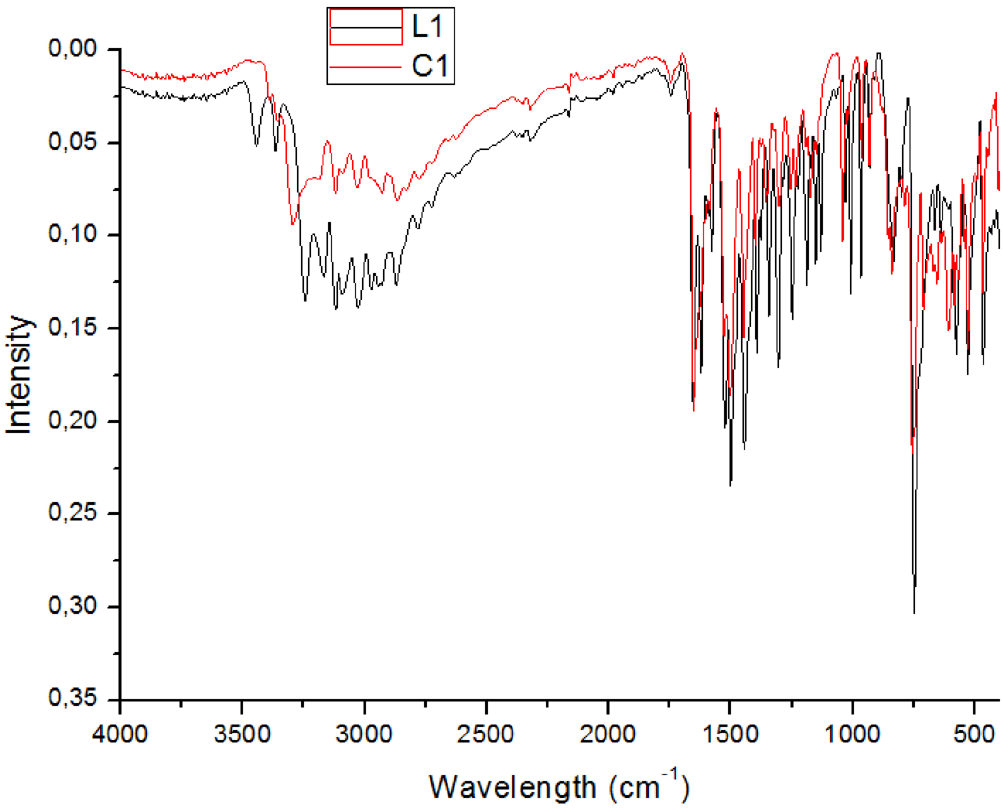

Figure S7. FT-IR comparison plot of $\boldsymbol{C_1}$ and $\boldsymbol{L_1}$.

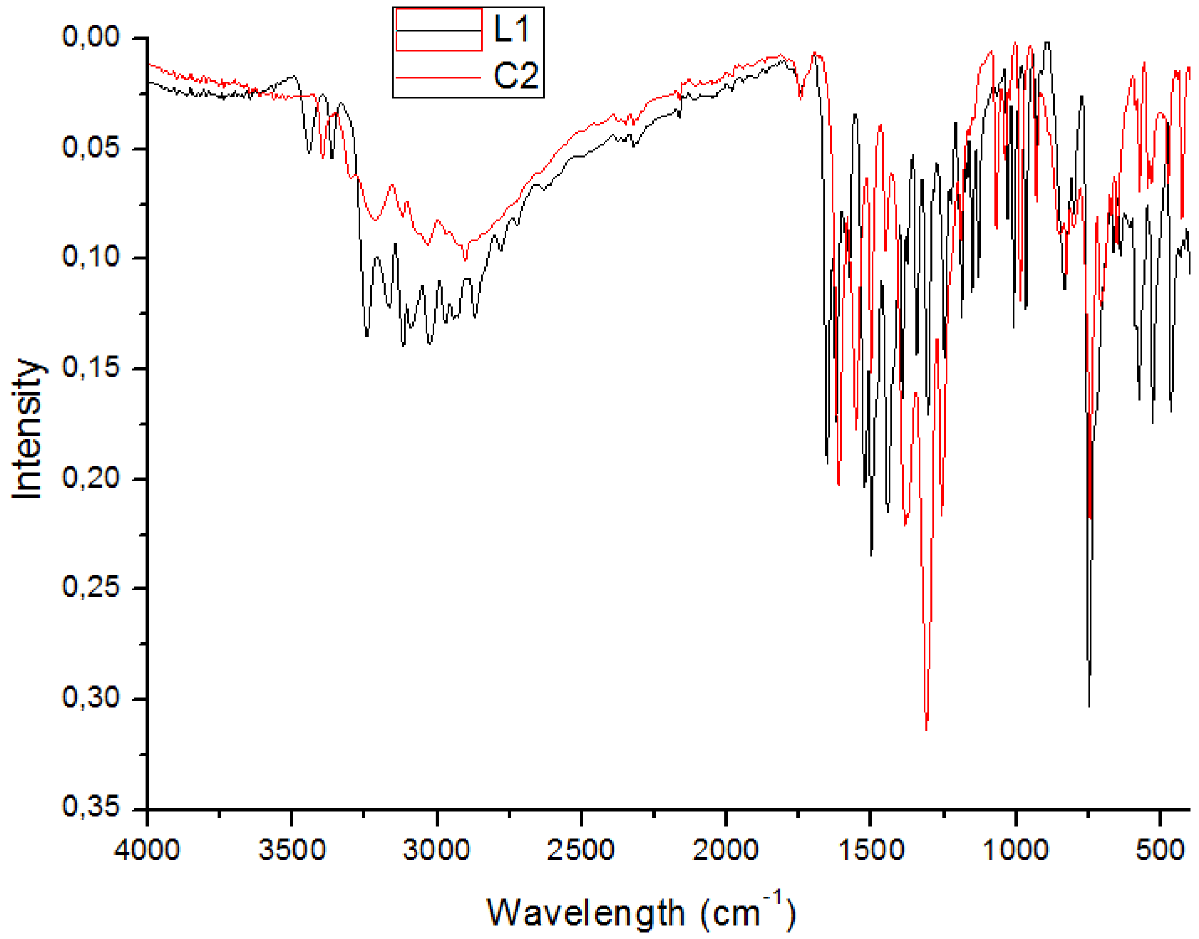

Figure S8. FT-IR comparison plot of $\boldsymbol{C_2}$ and $\boldsymbol{L_1}$.

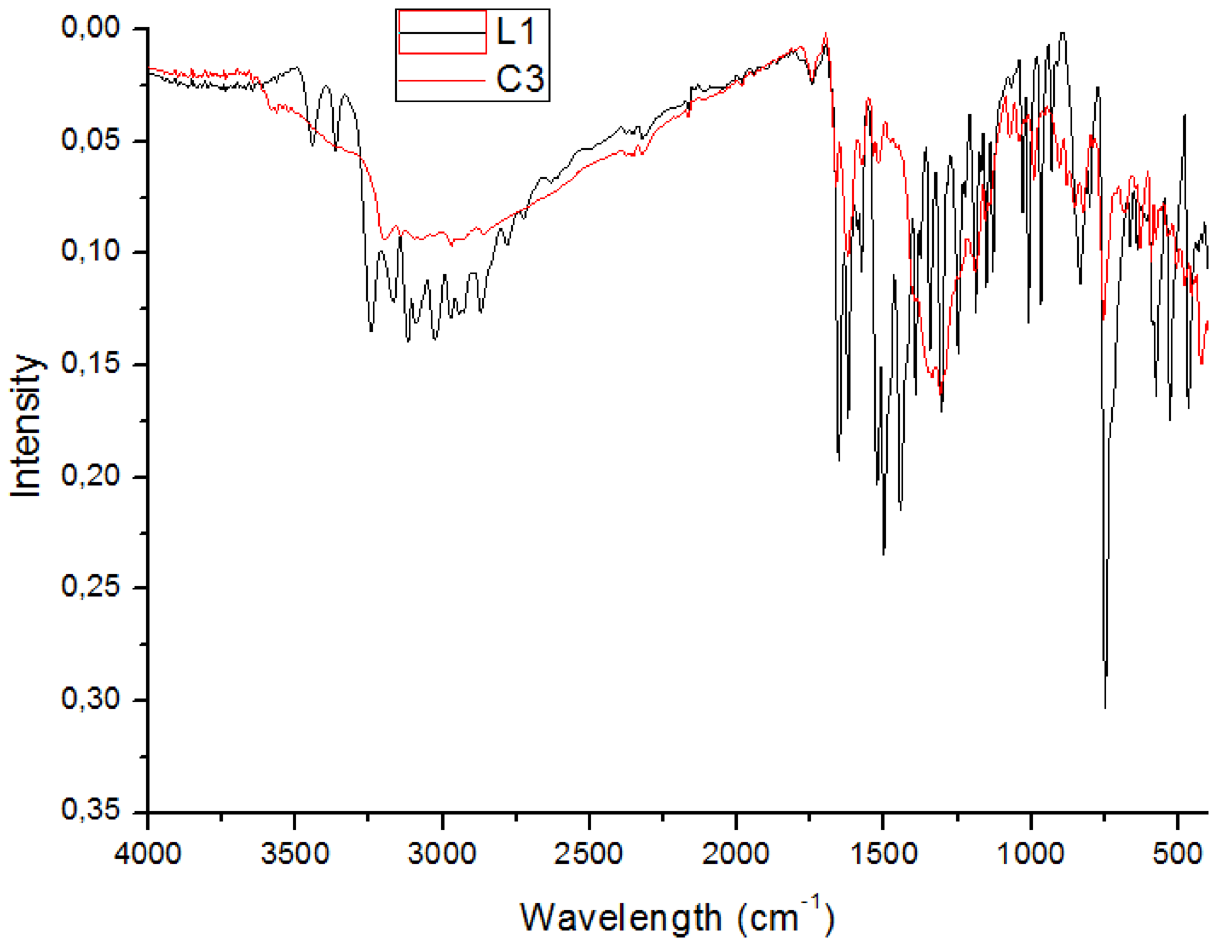

Figure S9. FT-IR comparison plot of $\boldsymbol{C_3}$ and $\boldsymbol{L_1}$.

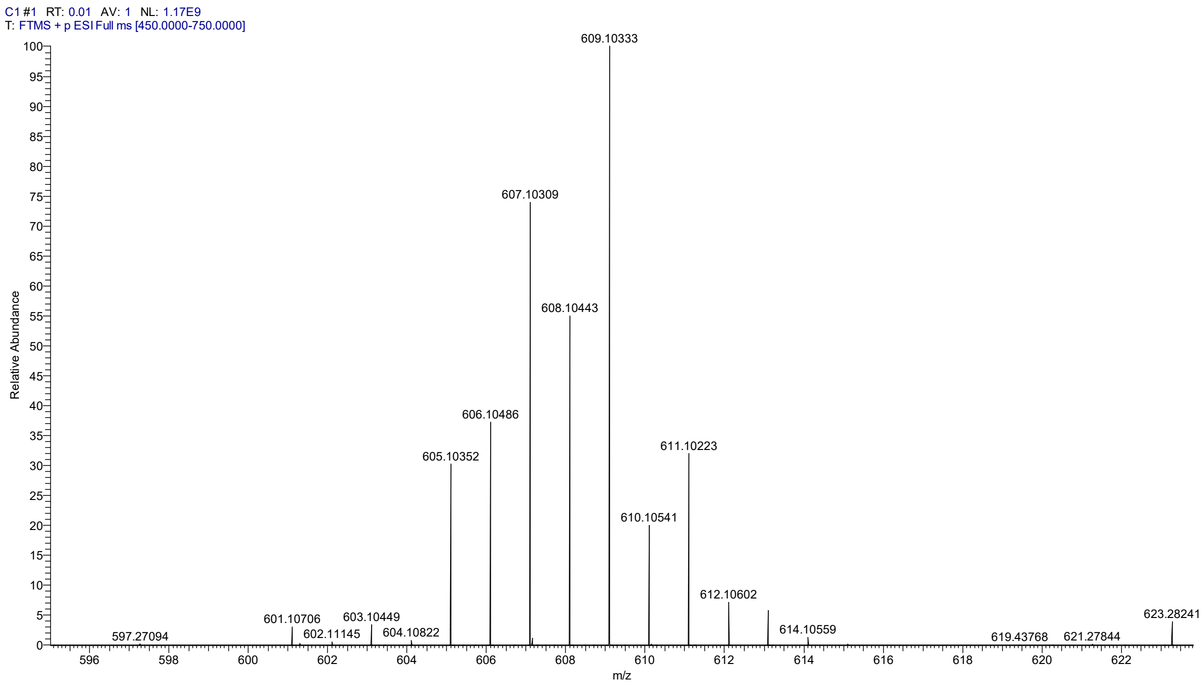

Figure S10. ESI-MS spectrum of $\boldsymbol{C_1}$.

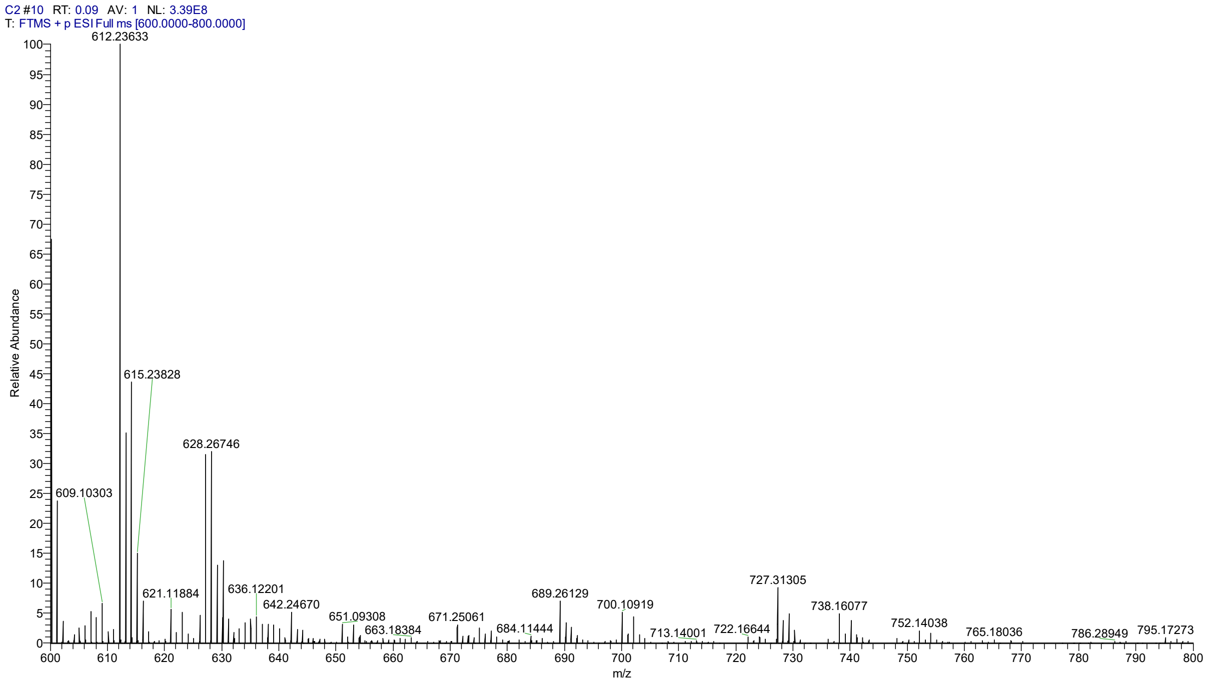

Figure S11. ESI-MS spectrum of $\boldsymbol{C_2}$.

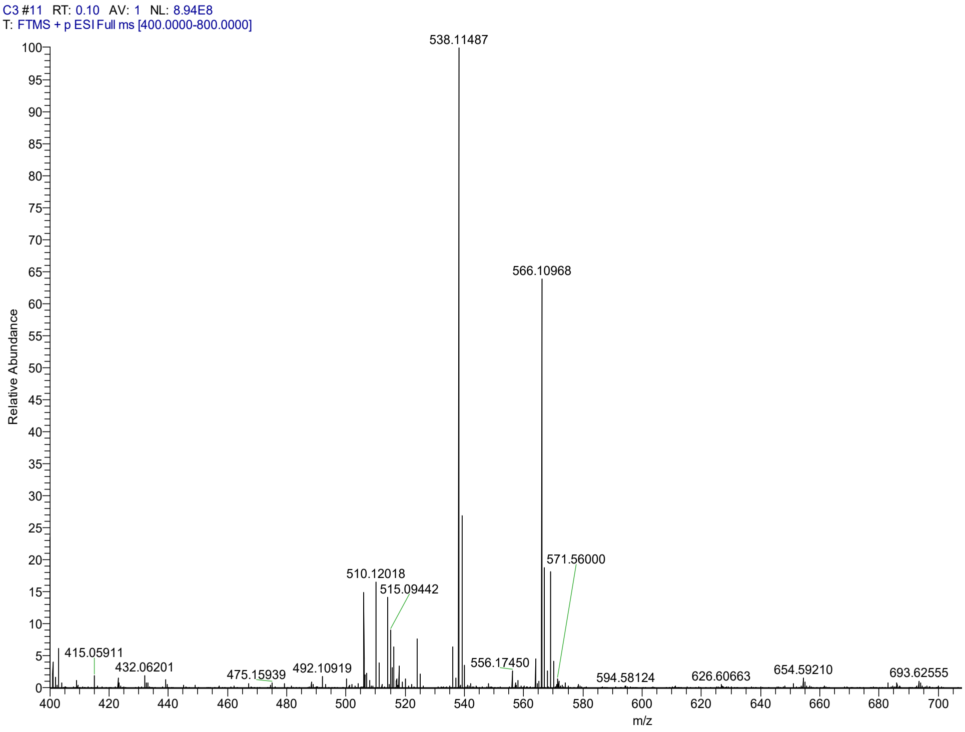

Figure S12. ESI-MS spectrum of ***C_3***.

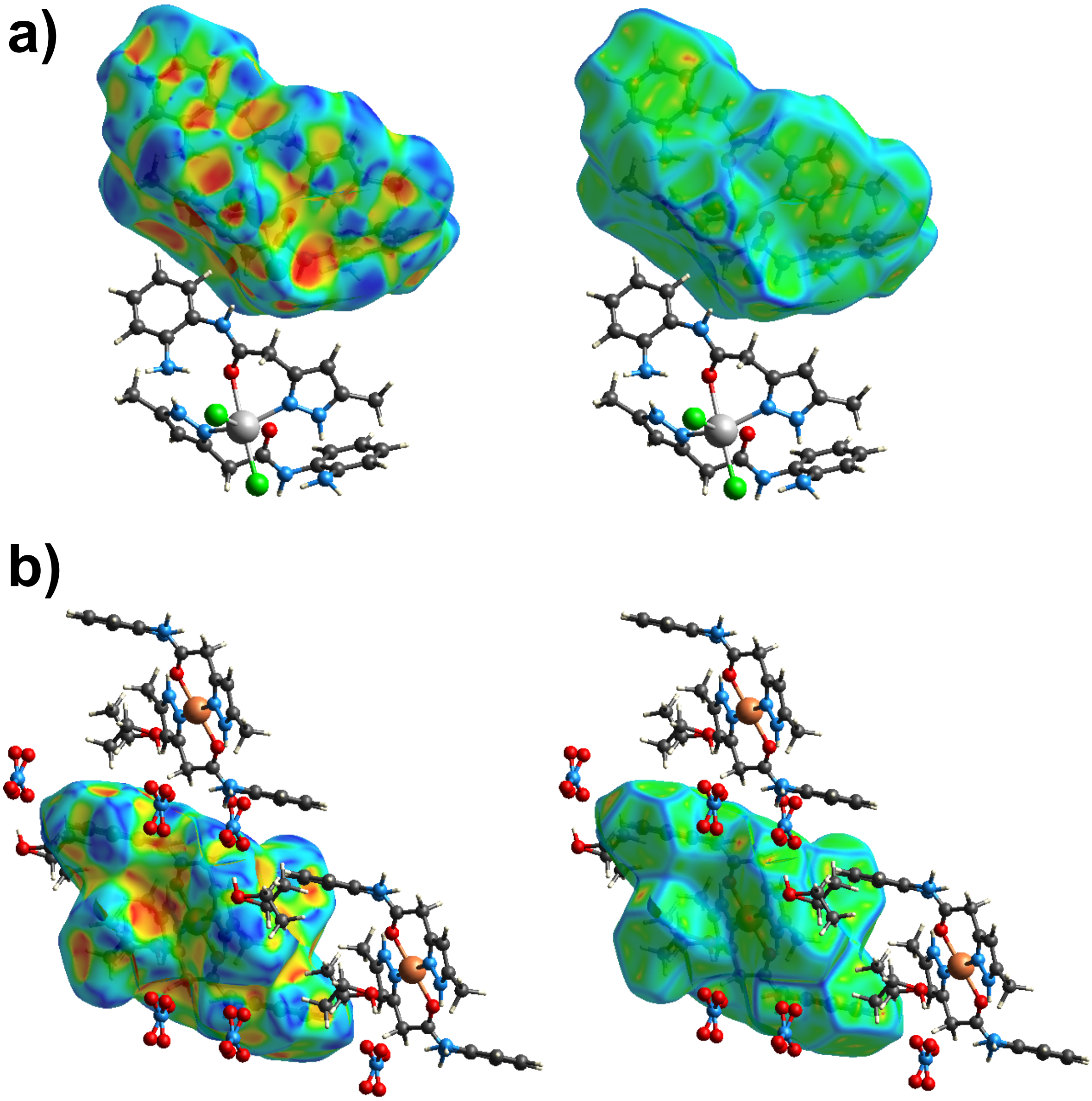

Figure S13. shape index (shown in left side) and curvedness (shown in right side) of $\boldsymbol{C_1}$ (a) and $\boldsymbol{C_2}$ (b).

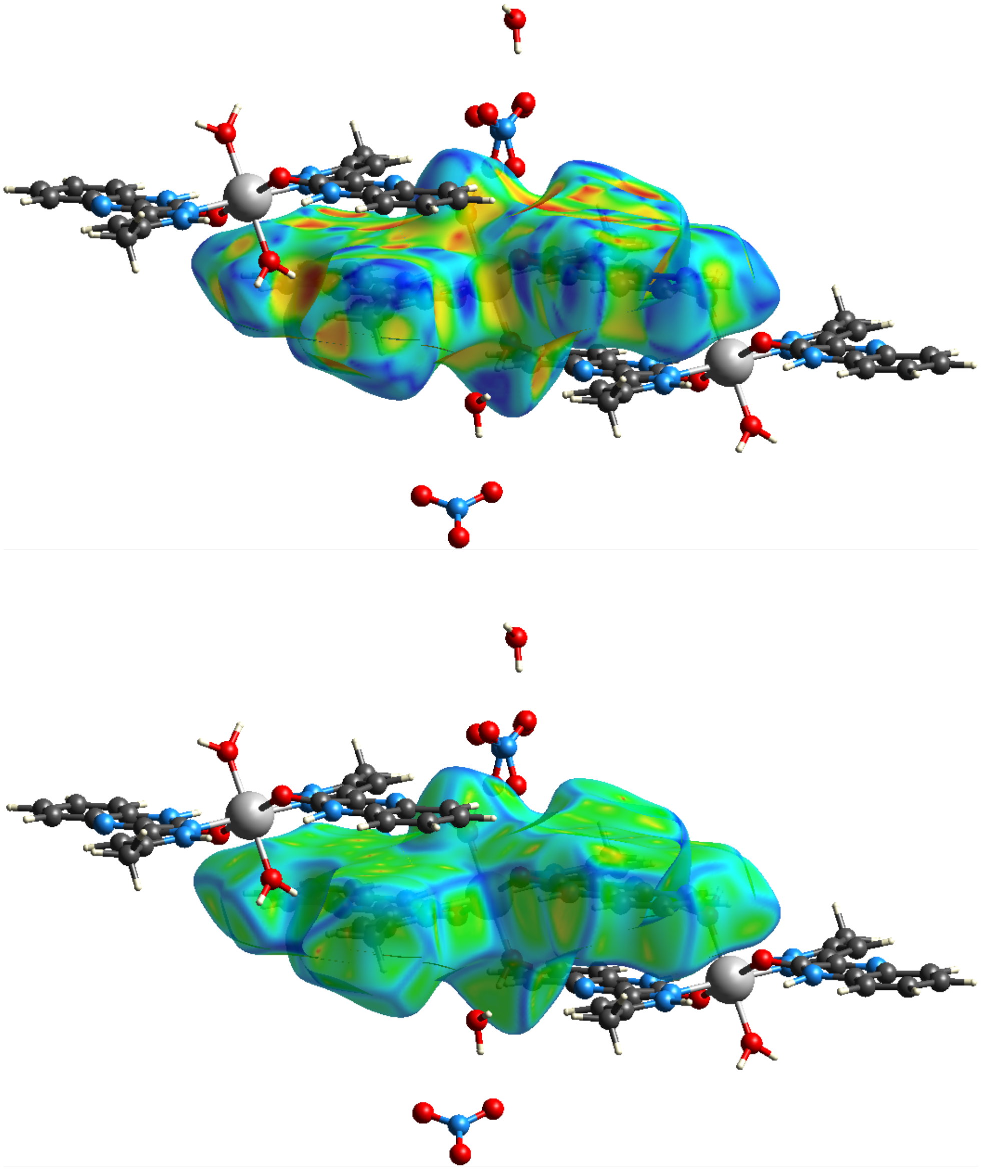

Figure S14. Shape index (*top*) and curvedness (*bottom*) of ***C*$_3$**.

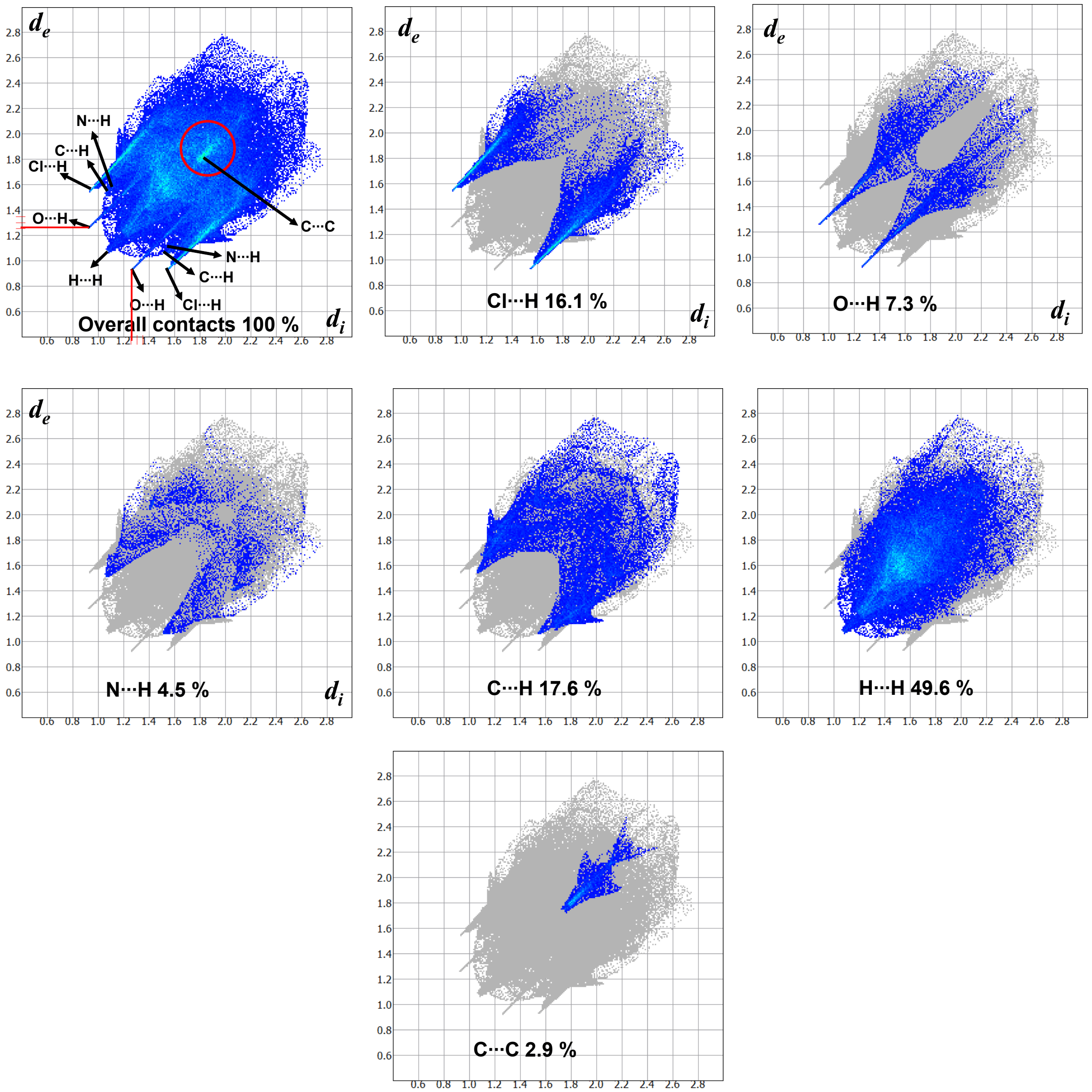

Figure S15. 2D Fingerprint plots derived from Hirshfeld surfaces, displaying various intermolecular interactions for $\boldsymbol{C_1}$.

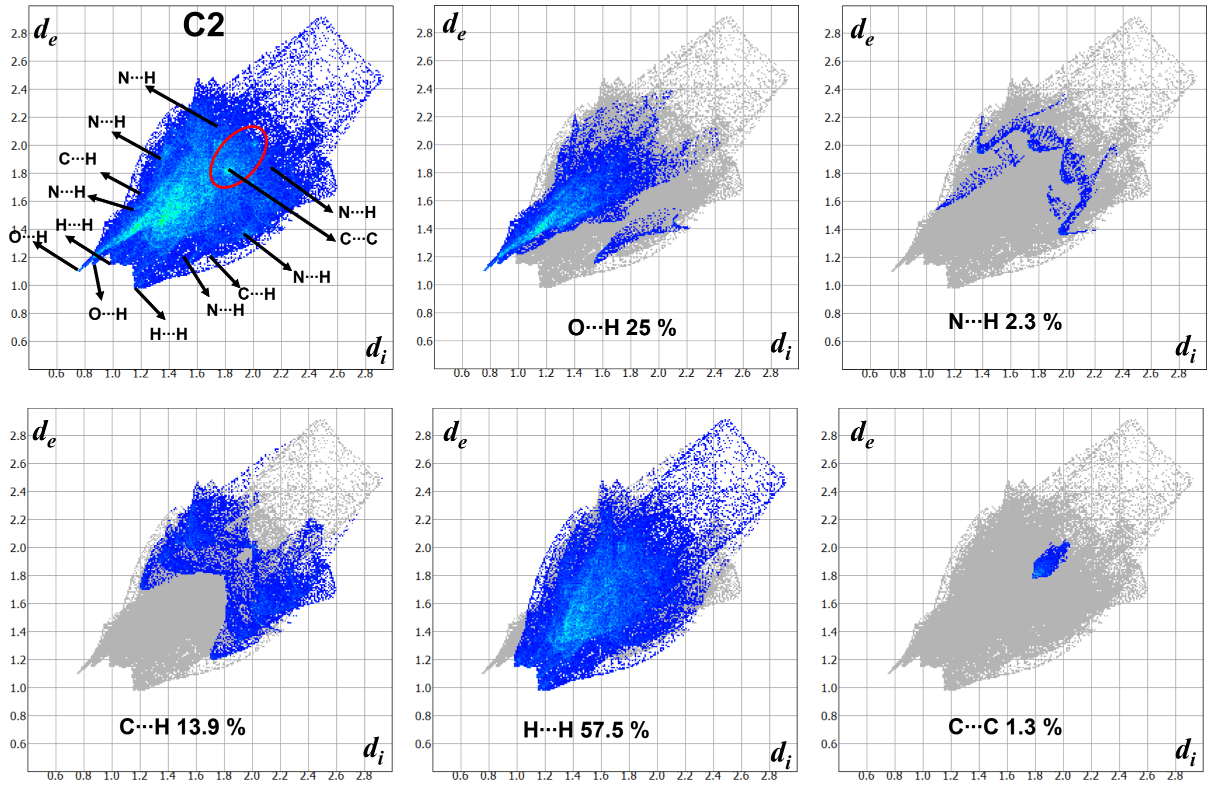

Figure S16. 2D Fingerprint plots derived from Hirshfeld surfaces, displaying various intermolecular interactions for ***C*$_2$**.

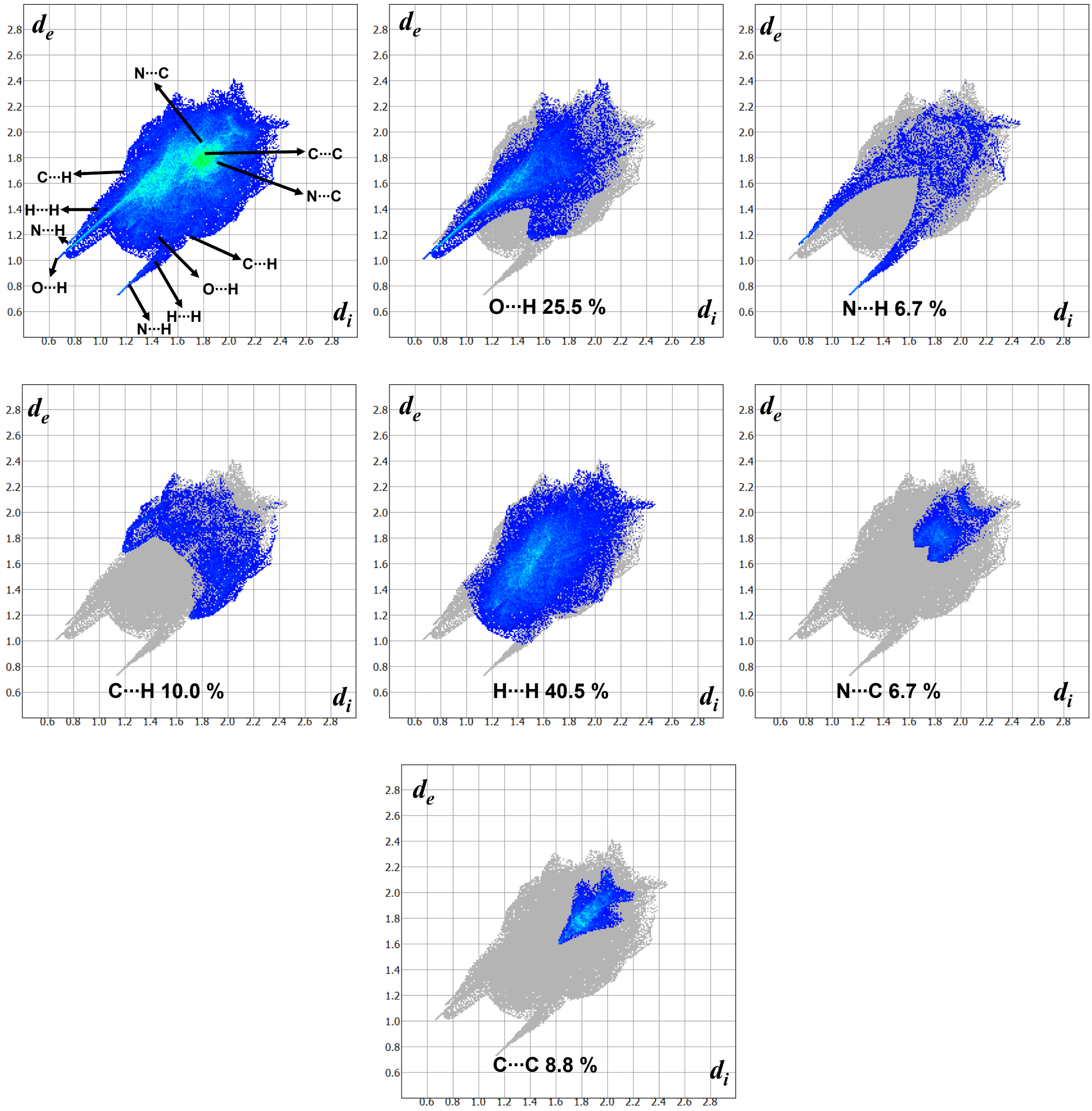

Figure S17. 2D Fingerprint plots derived from Hirshfeld surfaces, displaying various intermolecular interactions for $\boldsymbol{C_3}$.
